# DNA Aptamers Block the Receptor Binding Domain at the Spike Protein of SARS-CoV-2

**DOI:** 10.3389/fmolb.2021.713003

**Published:** 2021-08-12

**Authors:** Fabrizio Cleri, Marc F. Lensink, Ralf Blossey

**Affiliations:** ^1^ University of Lille, CNRS UMR8520 IEMN, Institut d’Electronique, Microélectronique et Nanotechnologie, Lille, France; ^2^ University of Lille, Departement de Physique, Villeneuve d’Ascq, France; ^3^ University of Lille, CNRS UMR8576 UGSF, Unité de Glycobiologie Structurale et Fonctionnelle, Lille, France

**Keywords:** DNA aptamers, SARS-CoV-2, spike protein, molecular dynamics, angiotensin converting enzyme-2, free energies

## Abstract

DNA aptamers are versatile molecular species obtained by the folding of short single-stranded nucleotide sequences, with highly specific recognition capabilities against proteins. Here we test the ability of DNA aptamers to interact with the spike (S-)protein of the SARS-CoV-2 viral capsid. The S-protein, a trimer made up of several subdomains, develops the crucial function of recognizing the ACE2 receptors on the surface of human cells, and subsequent fusioning of the virus membrane with the host cell membrane. In order to achieve this, the S1 domain of one protomer switches between a closed conformation, in which the binding site is inaccessible to the cell receptors, and an open conformation, in which ACE2 can bind, thereby initiating the entry process of the viral genetic material in the host cell. Here we show, by means of state-of-the-art molecular simulations, that small DNA aptamers experimentally identified can recognize the S-protein of SARS-CoV-2, and characterize the details of the binding process. We find that their interaction with different subdomains of the S-protein can effectively block, or at least considerably slow down the opening process of the S1 domain, thereby significantly reducing the probability of virus-cell binding. We provide evidence that, as a consequence, binding of the human ACE2 receptor may be crucially affected under such conditions. Given the facility and low cost of fabrication of specific aptamers, the present findings could open the way to both an innovative viral screening technique with sub-nanomolar sensitivity, and to an effective and low impact curative strategy.

## 1 Introduction

At the end of 2019, a novel virus belonging to the coronavirus family has been identified, initially in the population of the Chinese city of Wuhan. Since then, the virus has practically spread across the whole world, requiring drastic measures both for treatment of the patients and to avoid uncontrolled spreading of the disease among the human population. This virus has been designated SARS-CoV-2 by the Coronavirus Study Group (CSG) of the International Committee on Taxonomy of Viruses. Coronaviruses are enveloped viruses, their protein capsid being decorated by club-shaped glycoprotein spikes (S-protein) that protrude from the surface, as it is the case of, e.g., SARS and MERS viruses (Xu et al., 2020). However, this novel coronavirus is still distinct from both SARS and MERS, with multiple mutations identified in different genomic regions (Lu et al., 2020a). The surface-covering S-proteins allow the virus to bind to certain receptors on human cells, such as the widely distributed ACE2. Like other members of the same family, SARS-CoV-2 carries a positive-sense, single-stranded RNA genome belonging to the Coronaviridae family, with about 70% similarity in genetic sequence to SARS. The characteristic structure of its S-protein is made up of three protomers, each including two key domains, S1 and S2. S1 with its receptor-binding subdomain (RBD) is required for host-cell receptor binding, and S2 is required for membrane fusion (Walls et al., 2020; Wrapp et al., 2020; Yan et al., 2020; Lan et al., 2020). Because of its steric prominence, the S-protein is one of the main targets for both molecular-based therapy and screening of the virus.

Current anti-viral screening methods mostly analyse throat and nose swab samples with RT-PCR, which uses nucleic acids as target, or serologic blood samples and IgM/IgG biomarkers. The diagnostic accuracy of RT-PCR highly depends on the “virus-specific diagnostic window”, and the analytical sensitivity of this assay is potentially plagued by false SARS-CoV-2 negativity, attributable to the low viral loads especially in asymptomatic or mildly symptomatic patients. Despite the large acceptance of these assays, they are expensive and time consuming. On the other hand, serologic tests are based on recognition of antibodies; however, IgM have little specificity since they are active for about any kind of viral infection that may have attacked the organism, and the more specific IgG arise only several weeks after the infection thus being of little help for the early detection. Also “rapid” antigenic tests have been developed, which recognize parts of the virus proteome, however with a reduced sensitivity compared to PCR- and antibody-based tests (Smithgall et al., 2020). Given the highly transmissible nature of this virus, its relatively high fatality rate, and the rapid development of many virus variants across the infected populations, there is urgent need for highly specific, early-stage and selective testing, massively available, easily adaptable to variants, and at the lowest possible cost.

Aptamers are artificial oligonucleotide or peptide molecules that bind to a target molecule with high specificity. Aptamer-protein-based analytical methods have become popular in the last years. Just like antibodies, aptamers are capable of binding a target, and also of modulating or blocking its activity. Generated by an *in vitro* selection process from pools of random sequence oligonucleotides [the SELEX technique, see e.g. Famulok and Mayer (2014); Darmostuk et al. (2015)], targeted aptamers have already been produced for hundreds of different protein targets. A typical aptamer is 10–30 kDa in size (about 30–60 nucleotides), it binds its target with sub-nanomolar affinity and, most importantly, can discriminate against closely related targets. Structural studies indicate that aptamers are capable of using the same types of binding interactions that drive affinity and specificity in antibody-antigen complexes. Aptamers of various type have been already identified and tested in the anti-viral domain in recent years. For example, Cheng et al. (2008) found that 5 pg/*μ*L of their ssDNA aptamer could effectively stop replication of H5N1 avian-influenza virus; (Jang et al., 2008) demonstrated an efficient SARS-helicase activity inhibition by a RNA aptamer; recently, (Song et al., 2020) identified two candidate ssDNA aptamers that seem to bind efficiently to the RBD of the S-protein of SARS-CoV-2; in another recent study (Chen et al., 2020), DNA aptamers were shown to be able to efficiently recognize the SARS-CoV-2 nucleocapsid protein.

In the present work, we investigate by means of state-of-the-art protein docking and large-scale molecular dynamics simulations, the interaction of the two experimentally identified DNA aptamers (Song et al., 2020) with the S-protein of SARS-CoV-2. Our initial purpose was to characterize the affinity of the aptamer for the binding domain of the S-protein, in support of the use of aptamers as fast and efficient anti-viral screening. However, an even more interesting question concerns the detailed molecular interaction between aptamers and the viral protein(s). Indeed, it could be possible that these same aptamers may block, or at least considerably slow down, the transition of the S1 domain from the closed to the open conformation, thereby blocking the access of the cell surface receptors to the virus surface. In this work we will focus on this key aspect, showing that the DNA aptamers, while binding very efficiently to the designated RBD on one protomer of the S-protein, as shown in the experiments, also form and maintain stable bonds with other subdomains of adjacent protomers. This extended bonding creates a sort of “bridge”, which results in hampering the opening of the RBD to the cell receptors. By means of extensive MD simulations on the two experimentally identified aptamers, we could characterize the nature and strength of the aptamer-protein interactions, mainly hydrogen bonds complemented by non-covalent, long-range interactions. Further umbrella sampling simulations of protein configurations going from closed-to open-RBD, with and without the DNA aptamer attached, also allowed to characterize the large variations in free-energy barriers; this, in turn, permitted to set a relative scale of the announced blocking effect. Finally, simulations of docking of the human ACE2 receptor to the S-DNA complex, demonstrated that the RBD is strongly affected by the presence of the DNA aptamer, and may lead to a drastic reduction of the cell receptor binding efficiency. Once such predictions would be experimentally validated, DNA aptamers could contribute an alternative, low-cost and low-impact therapy, apt to reduce the virus efficacy in the host organism. Virtual screening of DNA aptamers by computer simulation could, moreover, quickly and cheaply adapt to rapidly mutating viral targets, as well as to new Coronavirus-family strains that could appear in the future.

## 2 Methods

### 2.1 Molecular Structures of the S-Protein and Angiotensin Converting Enzyme-2

**FIGURE 1:**
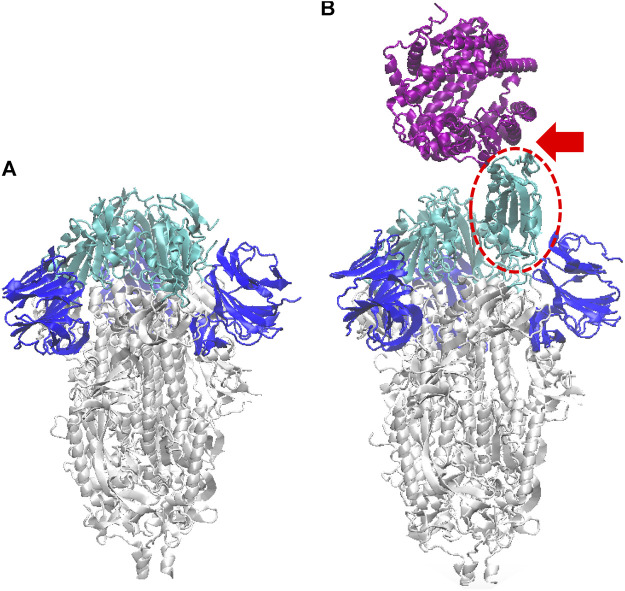
Ribbon model of the SARS-CoV-2 S-protein (glycans omitted for clarity), in the closed configuration **(A)**, and with one monomer open **(B)** (dashed red oval). Here and in the following figures, the S1-RBD subdomain of each monomer is depicted in cyan, and the N subdomain in blue. In **(B)** also the ACE2 human receptor is represented (purple), interacting with the S1 domain in open configuration (see red arrow); atomic structure obtained by aligning the pdb 6VSB (Wrapp et al., 2020), with the co-crystallized S-protein RBD and ACE2 structure, pdb 6M0J (Lan et al., 2020).

The S-protein is a homologous trimer, with each protomer being composed of the two domains S1 and S2, and a transmembrane region. We ran a series of simulations for a glycosylated model of the S-protein, from the theoretical configurations made available by the group of R.J. Woods (Grant et al., 2021). All these glycoforms are based on the PDB entry 6VSB from the RCSB Data Bank (Wrapp et al., 2020), reporting the experimental pre-fusion conformation of the S-protein with one protomer “open”, and integrated by glycomics data. Given the ample variability of the N-glycans observed on the S-protein experimental configurations (Casalino et al., 2020; Walls et al., 2020; Woo et al., 2020; Grant et al., 2021), we adopted a “worst case” configuration, by choosing the homogeneous model with the longest glycan chains, namely the M9 composed by a 3-mer stem (GlcNAc–GlcNAc–3,6Mannose) and three branched mannose chains; 18 glycans are attached to each protomer, for a total of 54 glycosylation sites. The “closed” form of the protein, required for the interaction with the DNA aptamer, was reconstructed by copying one of the closed protomers and shifting it, to replace the open protomer of the original configuration (Figure 1); the non-glycosylated structure 6VXX with all the three closed protomers was used as template, for aligning the shifted protomer with the TMalign utility program (Zhang and Skolnick, 2005). The PDB structures were passed through the pdb2gmx utility of the GROMACS package, to assign hydrogens to the residues and write a full topology of the system. For the thermal equilibration simulations, the protonation state of histidines was automatically selected based on the closest possible hydrogen bonds; for the umbrella sampling simulations instead we had to impose a unique choice to all frames (see below), in order to maintain the same protein structure, therefore we arbitrarily imposed single protonation at the ND nitrogen. For the sake of comparison, we include also a series of simulations that were originally run on the PDB entries 6VXX and 6VYB (Walls et al., 2020), as reference for the non-glycosylated form of SARS-CoV-2 S-protein, in the closed and open forms, respectively. In the following, we label FG the fully-glycosylated model, and NG the non-glycosylated model.

The ACE2 human receptor molecular configuration was taken from the 6M0J entry (Lan et al., 2020). Although ACE2 is observed to dimerize *in vitro* (Yan et al., 2020), the interaction with the S-protein is likely to occur via only one monomer, given the large steric hindrance of both structures. Therefore, the monomeric structure of ACE2 was retained for the last part of our study.

### 2.2 Molecular Structure of Candidate DNA Aptamers

We took the sequences of the two candidate ssDNA aptamers from the recently published study by Song et al. (2020). These were extracted by a SELEX procedure of 12 rounds, over a pool of several millions random sequences directed against the RBD fragment of the S-protein. After reduction of redundant fragments, the two best candidates sequences are a 51-bp (**apta1** in the foregoing) 5′-CAG​CAC​CGA​CCT​TGT​GCT​TTG​GGA​GTG​CTG​GTC​CAA​GGG​CGT​TAA​TGG​ACA-3′, and a 67-bp (**apta2**) 5′-ATC​CAG​AGT​GAC​GCA​GCA​TTT​CAT​CGG​GTC​CAA​AAG​GGG​CTG​CTC​GGG​ATT​GCG​GATATGGACAC GT-3’.

For each sequence, (**apta1** and **apta2**), we obtained the 2D structure by the mfold web-server (Zuker, 2003); a double-check of the structures with NUpack (Zadeh et al., 2011) confirmed the geometries, with minor differences in the values of free energy. Supplementary Figure S1 in the Supplementary Material gives details of the 2D structures, which match those already obtained by Song et al. (2020). Supplementary Table S1, S3 also give the associated folding free-energy estimated on the basis of the nearest-neighbor model Zuker and Jacobson, 1998; it is readily appreciated that the main negative contributions to the 2D-folding Δ*G* come from the paired helices, while the main positive contributions come from the (more or less large) hairpin loops. Since there are no programs available to directly fold DNA, to obtain the 3D structures we firstly changed the thymines to uracil, in the 2D sequences written in Vienna format, and ran each structure with the RNAcomposer web-server (Popenda et al., 2012); then, uracil bases were reverted back to thymine simply by dropping the O2’ oxygen. Such a procedure, similar to the protocol proposed by Jeddi and Saiz, 2017, may induce minor variations in the structure, which were healed with a subsequent energy minimization step (see below). The final relaxed 3D structures will be used as starting point for the subsequent molecular studies.

It may be noted that the 3D conformations of the aptamers are deduced based on a two-step process, in which the secondary structure is firstly minimized on the basis of the simple nearest-neighbor interaction model, and then fed into a 3D model building program: as such, there is no guarantee that the lowest-energy structures selected in the first step would remain at the lowest energy also in the second step, followed by energy minimization, which implies a substantial contribution of elastic energy, long-range and dihedral interactions. Secondly, the stereochemical docking of the aptamer to the protein domains is also subject to a considerable uncertainty, as different methods and codes are known to give somewhat different results. For both these issues, the substantial convergence of the results obtained for the NG and FG structures constitutes a minimal proof of internal consistency.

Docking of the aptamers to the S1 domain of the S-protein in the closed conformation was performed by the HADDOCK web-server (van Zundert et al., 2016), separately for the NG and FG structures. In both cases, the protein structure was restricted to a S1 fragment of one single protomer, residues 1–700 (however much larger than the single RBD subdomain used in the experiments). For each sequence, we firstly explored several dockings with a small number (15–17) of DNA nucleotides as target, up to spanning the whole sequence; and secondly, a random docking in which the whole DNA was used as target. A large number of docked structures with very close energies were produced by HADDOCK. We selected the best (lowest-energy, best Haddock score) configuration for the **apta1** and **apta2**.

**FIGURE 2:**
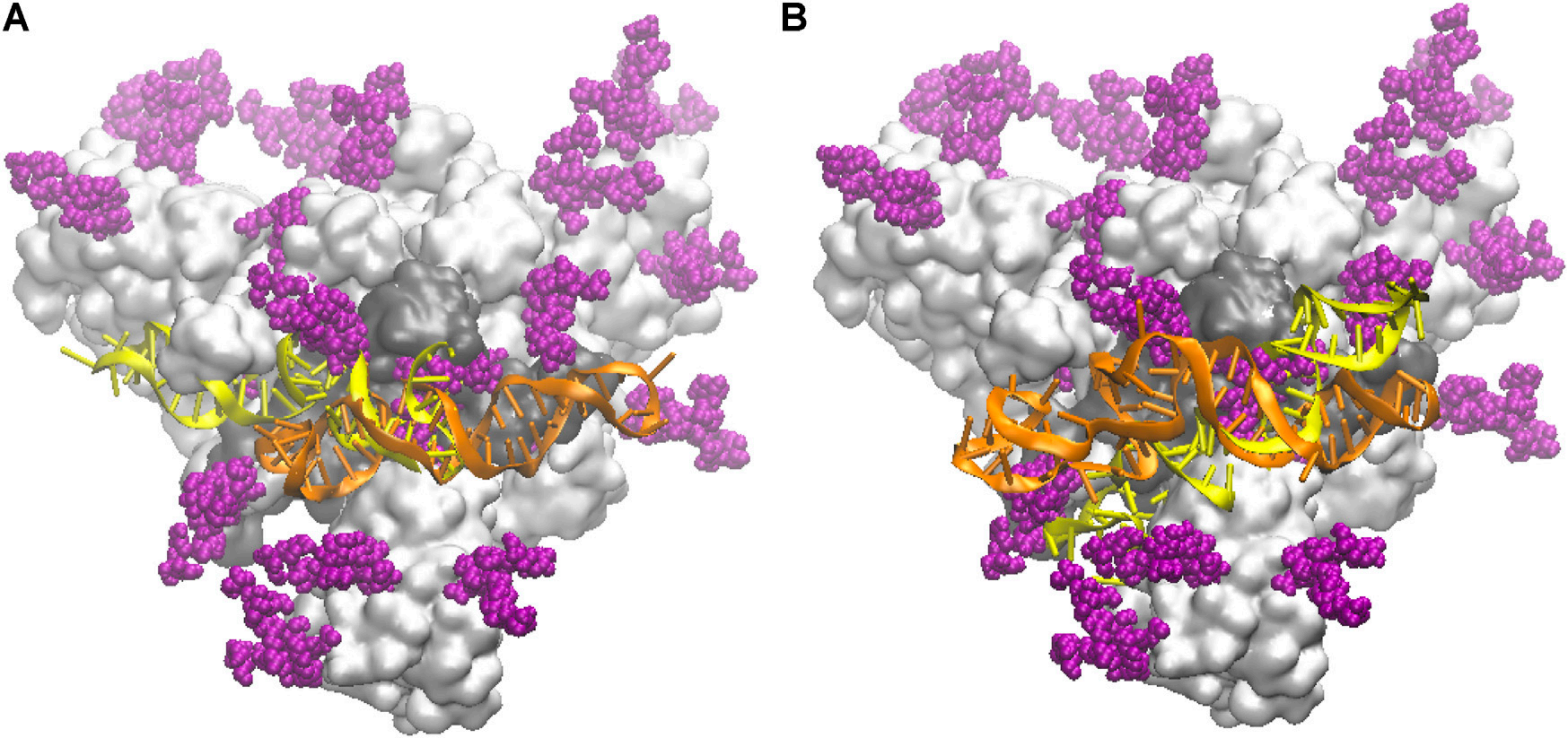
Schematic of the initial docked DNA/S-protein structures from HADDOCK. The trimer head of the S-protein is shown as a light grey surface, slightly tilted back with respect to the main (central) axis for better view; the RBD in contact with DNA aptamers is the dark grey area; glycans are depicted by purple spheres; the DNA conformations are superposed, in yellow the one for the NG protein, in orange the one for the FG protein. **(A)** The shorter **apta1** DNA bound to the S1-RBD domain. **(B)** The longer **apta2** bound to the S1-RBD domain.

In Figure 2 we compare the docked configurations for the NG and FG protein structures, in contact with both the smaller apta1 (left panel) and the longer apta2 (right panel). For the **apta1**, the pose of the DNA turns out to be flipped by 180° and mirror reflected, between the NG and FG structures (yellow and orange DNA, respectively, in Figure 2A). By this inversion, the part of DNA in direct contact with the protein (nucleotides 44–49) remains the same, in particular the H-bond between LYS356 and A49 is common to both the NG and FG; the latter also makes a second H-bond between THR470 and G25. For the longer **apta2**, the DNA covers approximately the same position in the NG and FG protein (Figure 2B), however with some relative deformation due to the presence of the glycans in the FG. The initial H-bond network is also different, the key residues implicated being THR345, SER349, ARG357 in the NG, compared to THR470, CYS488, ARG509 in the FG; for the latter, also some H-bonds between DNA and glycans are identified (see discussion in *Binding of DNA Aptamers to the S-Protein* below). While such differences highlight the relevance of the glycan shield in setting the interactions of the S-protein (Casalino et al., 2020; Grant et al., 2021), it should be noted that the docking configurations are just starting points for the subsequent MD simulations, which may end up with quite different bonding structures after long thermal equilibration.

### 2.3 Molecular Dynamics Simulations

For the FG simulations we adopted the CHARMM-36 database (MacKerell et al., 1998; Foloppe and MacKerell, 2000), which readily includes a well-tested set of parameters for all the glycan structures (Mallajosyula et al., 2015). However, for the earlier NG simulations the AMBER99 force field database (Ponder and Case, 2003; Cheatham and Case, 2013) with the BSC1 extension for nucleic acids (Pérez et al., 2012), were used for the molecular bonded and non-bonded force parameters. The two descriptions are largely equivalent in most respects (see e.g., Fadda and Woods (2010)), the choice is just a matter of convenience, the glycan dataset being already included in the CHARMM library with no need for further adaptation.

For all the molecular dynamics (MD) simulations we used the GROMACS 2020 computer code package (Berendsen et al., 1995; Lindahl et al., 2001). For the thermal stability study, the ensemble of the complete S-protein and DNA aptamers were solvated in a water box of size 23 × 23 × 23 nm^3^ with periodic boundary conditions in the three directions, containing about 380,000 TIP3P water molecules, plus Na^+^, Cl^−^ and Mg^2+^ ions to ensure neutralization of the phosphate backbone charge, at a physiological concentration of 0.1 M NaCl and 0.005 M MgCl_2_. Similar conditions were used for the umbrella sampling and force-driven studies of Section 3.2 and Section 3.3, but with a smaller water box of 14 × 14 × 18 nm^3^ and NaCl ions only. All the production MD runs were carried out at the temperature of 310 K and pressure of 1 atm. The low-mass, N-bonded glycans added to the experimental protein structures (Walls et al., 2020) were removed for the MD simulations of the NG structure.

Coulomb forces were summed with particle-mesh Ewald sum, using a real-space cutoff of 1.2 nm (equal to the cut-off radius of shifted Van der Waals potentials). We used rigid bonds for the water molecules, with a time step of 2 fs for the thermal equilibration phases and 1 fs for production and force-pulling runs. For the thermal stability study, preparatory runs at constant-{*NPT*} and temperatures increasing in steps of 100 K from T = 10 to T = 310 K lasted 20 ns, and were followed by thermal stability simulations at constant-{*NVT*}, which extended to 200 ns for each configuration. Statistics were accumulated over the last parts (100–150 ns) of each trajectory.

For the umbrella sampling and potential-of-mean-force (PMF) simulations we preferred not to use any of the many available free-energy sampling methods to obtain the lowest-energy path, because of the large size and complexity of our system, for which we study an ample hinge motion of the RBD (see e.g. the review by Orellana (2019)), and the discussion in the following Section. Instead, we reconstructed a putative opening path from the closed to the open conformations of the S1-RBD subdomain, by using the morph utility of the Chimera package (Pettersen et al., 2004). 50 intermediate frames were obtained along the shortest geometric path, at distances of 0.25 Å along this fictitious reaction coordinate, and the corresponding configurations were reconstructed (note that such a spacing is one order of magnitude smaller than usually assumed in PMF calculations). Then, the 50 conformations were geometrically realigned on the reference closed structure with TMalign, thereby obtaining 50 complete configurations of the S-protein, each with one single monomer transitioning from closed to open. After this “cold” reconstruction process, the 50 configurations were run through the pdb2gmx GROMACS utility and solvated in ionized TIP3P water (see above), in such a way to obtain strictly the same atom-ordered structures, with the same number of water molecules and ions, in order to represent the putative result of a MD trajectory along the closed-to-open transition. These 50 configurations were relaxed and equilibrated from 10K to 310 K in steps of 5 ns, and subsequently used in the umbrella sampling, with short (10 ns) force-constrained runs, to extract the potential of mean force (PMF) along the putative opening pathway. The final extraction of the free-energy profiles by weighted-histogram analysis (WHAM) was done with the GROMACS wham utility. The same protocol was repeated for all the docking configurations, by aligning on the reference structure the ensemble of the S1 and S2 subdomains carrying the docked DNA. For each new set of 50 frames, the whole procedure of thermal equilibration and force-constrained runs was repeated, and the potential of mean force was obtained.

Overall, the study used a total of about 1.2 million hours of CPU time, on 960–1,280 Intel CascadeLake cores +96 NVIDIA V100 GPUs of the IDRIS Jean-Zay supercomputer in Orsay, and on 504–1,008 Intel Broadwell cores of the OCCIGEN supercomputer in Montpellier, with typical running times of about 5 ns/h of wall-clock time. About 0.3 Terabyte of raw data were accumulated for subsequent post-processing.

## 3 Results

### 3.1 Binding of DNA Aptamers to the S-Protein

The results of 150-ns MD trajectories for the two aptamer configurations interacting with the S-protein trimer demonstrate a very stable bonding of each aptamer to the S1 domain of one single monomer of the whole protein. We extracted representative structures from the MD trajectories by the clustering algorithm of GROMACS. By looking at the centroid structures that collect most of the statistics (between 30 and 40% of the total trajectory), we observed that both aptamers make a number of hydrogen bonds with the S-protein, as detailed in the following.

**FIGURE 3:**
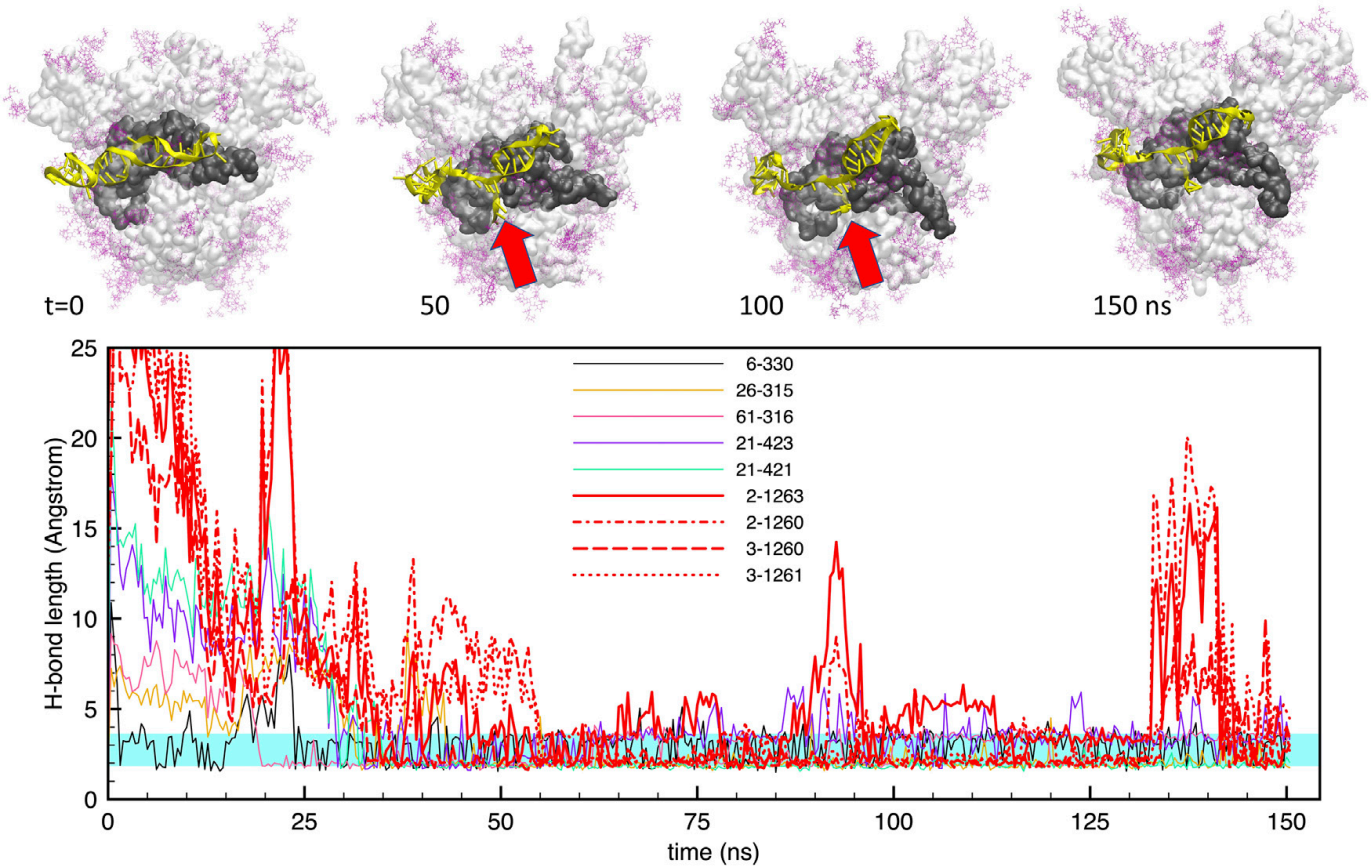
Evolution of the structure and hydrogen bonds formed by the DNA apta1 (51-nt) interacting with the S-protein trimer in the closed conformation. **Lower panel.** Time plot of the major H-bonds formed by nucleotides (numbers 1–51) and S-protein residues (numbers >300). Thin lines indicate the H-bonds between the aptamer and the RBD of monomer one; the thick red lines indicates the four extra H-bonds with the RBD of monomer 2 (times *t* ≃50–125 ns. The cyan shaded band indicates the typical interval of H-bond length (2.4–3.6 $\overset{{}^{\circ}}{A}$). **Upper panel.** Snapshots of the aptamer-S-protein contact, at times *t* = 0,50, 100, 150 ns DNA is depicted in yellow; protein surface in light grey, with the RBD of monomer one in dark grey; glycans in purple. The red arrows indicates the site of the extra H-bonds with the RBD of monomer 2. The atomic structures are tilted by about 30deg with respect to the central symmetry axis of the S-protein.

The interaction of the two aptamers with the FG structure of the S-protein, starting from the best docked configurations, revealed a strong adhesion of the aptamers at the S1-RBD subdomain, in broad agreement with the observations of Song et al. (2020). For the shorter **apta1**, we identified at least 5 H-bonds that were stable for more than 60% of the trajectory, and a number of less stable bonds, covering about 20–30% of the time. Figure 3 shows the time evolution of these H-bonds, together with some representative snapshots of the DNA-protein contact. It may be noted that, for the whole duration of the simulation, there are always at least 3–4 H-bonds keeping the aptamer in place. However, the conformation of the aptamer evolves substantially with respect to the initial docked structure. In particular, the 5’ end opens up, and penetrates within the interface between two adjacent protomers (red arrows in the figure). As we will describe below, this movement is chiefly linked to electrostatic interactions, and allows the aptamer to make further H-bonds (depicted by thick red lines in the lower panel of Figure 3); as a consequence, also the RBD conformation is distorted by such a strong interaction.

**FIGURE 4:**
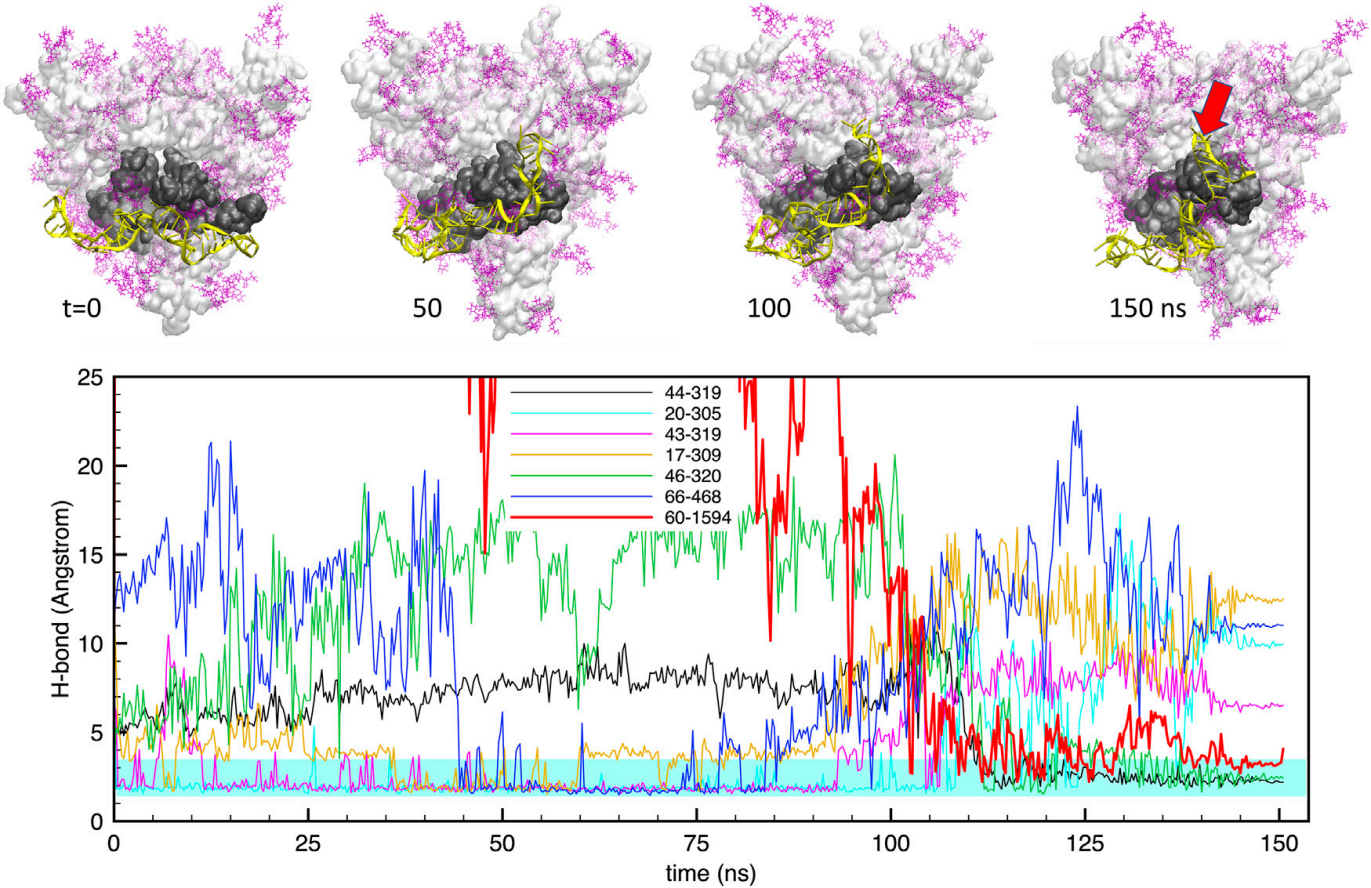
Evolution of the structure and hydrogen bonds formed by the DNA apta2 (67-nt) interacting with the S-protein trimer in the closed conformation. **Lower panel.** Time plot of the major H-bonds formed by nucleotides (numbers 1–67) and S-protein residues (numbers >300). Thin lines indicate the H-bonds between the aptamer and the RBD of monomer 1, the thick red line indicates the extra H-bond with the RBD of monomer 2 (setting in at times *t* > 100 ns The cyan shaded band indicates the typical interval of H-bond length (2.4–3.6 $\overset{{}^{\circ}}{A}$). **Upper panel.** Snapshots of the aptamer-S-protein contact, at times *t* = 0,50, 100, 150 ns DNA is depicted in yellow; protein surface in light grey, with the RBD of monomer one in dark grey; glycans in purple. The red arrow at *t* = 150 indicates the site of the extra H-bond with the RBD of monomer 2. All figures with the central axis perpendicular to the plane.

Similarly, Figure 4 shows the same data for the case of **apta2**. The situation is qualitatively similar, with a large number (up to 6) of H-bonds that maintain a stable bonding with the protein surface for the whole simulation. However, in this case we observe a large rearrangement of the aptamer structure after about 100 ns: starting from a docked configuration in which the DNA runs approximately parallel to the RBD, the aptamer evolves into a shape that “hugs” around the subdomain. This is clearly visible also in the H-bond plots in the lower panel, which show some bonds detaching and being replaced by others at around *t* ≃100 ns.

It has been recently reported (Nie et al., 2021) that small molecules with negatively charged groups, such as polysulphates, can bind to the S-protein via electrostatic interactions. The strong binding occurs in that case at the “cationic patch” of the RBD, namely ARG346, ARG355, LYS444, ARG466, and ARG509. While for apta2 the charged patch RBD remains practically hidden from the interaction, for the **apta1** we find GUA22 to make a stable interaction with LYS444, and CYT6 with ARG509; furthermore, we find the two phosphates of CYT4 and ADE5 to make a charge-charge contact with the ${NH}_{3}^{+}$ and ${NH}_{2}^{+}$ charges of LYS356 and ARG357. It appears, therefore, that the penetration of the 5’ tail at the interface between RBD and NTD (see above) should be largely helped by electrostatic interactions.

**FIGURE 5:**
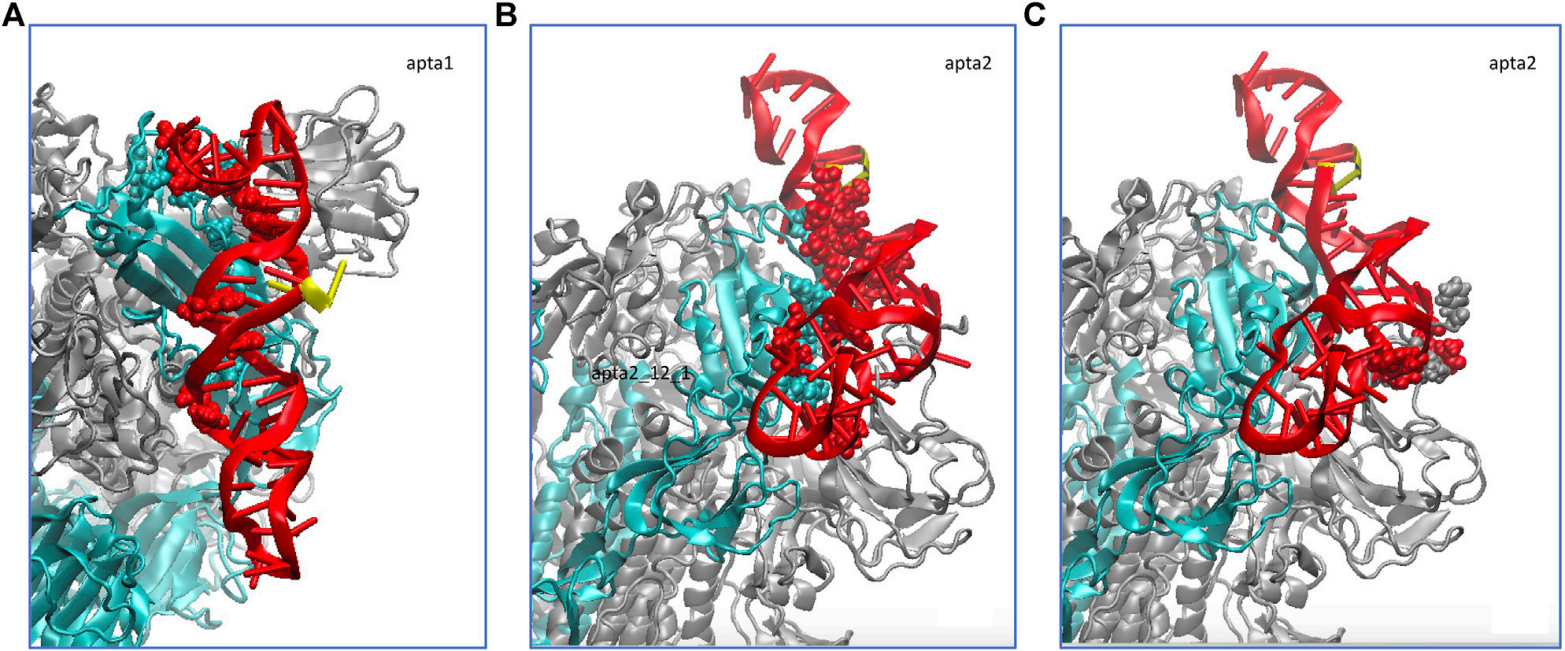
Schematic of the hydrogen bonds formed by the DNA aptamers (red ribbons) interacting with the non-glycosylated S-protein trimer in the closed conformation. **(A)** Binding of apta1 to the S1-RBD subdomain of monomer 1 (cyan ribbons). H-bonded residues are depicted with atomic spheres, cyan for the protein and red for the DNA; the 5′ and 3′ ends of the DNA are depicted in yellow. **(B)** Binding of apta2 to the S1-RBD subdomain of monomer 1. **(C)** Extra hydrogen bonds formed by apta2 with the N subdomain of the adjacent monomer 2.

We did a similar analysis also for a short 100-ns MD run of the NG S-protein, for the sake of comparison. The results are qualitatively similar to the FG, besides obvious differences in the atomic-scale details. Also in this case, the smaller aptamer **apta1** makes on average 10 hydrogen bonds with the S1-RBD domain, whereas the longer **apta2** makes about 11–12 strong hydrogen bonds, plus a number of lighter and fluctuating bonds. Figure 5 shows the average H-bonding configurations from the GROMACS cluster analysis, by representing with atomic spheres the interacting residues from the protein (cyan) and the DNA aptamers (red). For the longer apta2, a subset of 6 H-bonds, mostly arginine residues ARG346, ARG357 and ARG466, plus LYS356 and ASN450, are very stable in time, while the other five or six interactions are somewhat less stable and fluctuating.

Importantly, however, we also find that the DNA aptamer docked at the S1 domain of one of the protomers of the CoV-2 spike protein, also starts interacting with other subdomains of adjacent monomers. As shown in Figures 3, 4 above (see the thick red lines) a number of extra H-bonds are formed between each aptamer and one subdomain other than the RBD of monomer 1, to which each DNA was initially docked. In the case of **apta1**, a number of extra bonds are brought about by the 5’ end invading the N domain of protomer 2: notably, CYS166, THR167 and GLU169 of protomer two make not less than four extra H-bonds with thymine and cytosine in positions 2 and 3, for the largest part of the trajectory. In the case of **apta2**, one strong H-bond is made at THR500 of the RBD of protomer 2, plus a few less strong bonds, starting from the moment of the major change in aptamer conformation at time *t* > 100 ns. Such interactions constitute a sort of “bridge” between pairs of adjacent protomers, the DNA being strongly bound to the RBD of one, while crossing over to bind to a subdomain of the other. We will show in the next Section 3.2 how such a bridging may represent a considerable impediment to the opening of the S1, thereby radically changing the dynamics of the interaction of the viral S-protein with human cell receptors like the ACE2.

In the NG simulations, for the smaller apta1 such an extra interaction is limited only to exchange of long range forces (VdW and electrostatic) with a few flanking residues from a nearby protein monomer, whereas the longer apta2 is able as well to make new H-bonds with the N subdomain of a different protomer, adjacent to the one to which it was primarily attached. Up to four extra H-bonds are observed in this case (see Figure 5C, grey and red atomic spheres for protein and DNA, respectively); only extra H-bonds with occupancy of more than 50% along the entire MD trajectory were retained, and such extra bonds are very stable at occupancies between 60 and 90%.

It is worth noting that such bridging configurations of the DNA aptamers, covering pairs of adjacent protein monomers, could not have been expected on the mere basis of the experimental SELEX procedure (Song et al., 2020), which was performed in solution with just isolated monomer fragments of the RBD subdomain. Such a finding opens the way to a different interaction mode of the aptamers that, while binding to their target, can also interfere with the mechanical functioning of the S1-RBD opening mechanism and the subsequent receptor binding.

**FIGURE 6:**
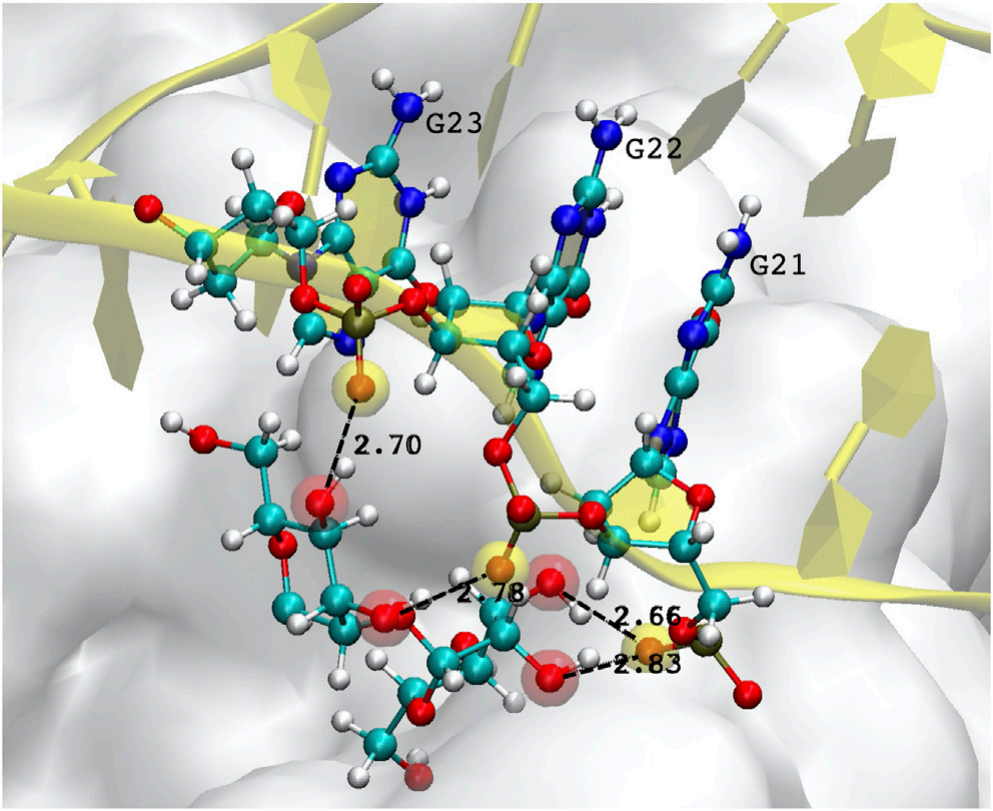
Example of hydrogen bonds formed by the DNA aptamer interacting with the glycans of the S-protein. In this case, three consecutive guanines form four bonds with two mannoses. Atoms participating in the H-bond are highlighted by a transparent red (mannose hydroxyl oxygen) or yellow sphere (DNA backbone oxygen).

A special mention should be reserved for the possibility of DNA-glycan contacts. This is uncharted territory, since there are no biological reasons for which DNA should interact with sugars, and the relative examples in the literature are therefore extremely scarce. Generally speaking, glycosylation is thought to occur in the endoplasmic reticulum and Golgi bodies, so that there are no natural occasions for DNA to come into contact with glycans. In the few studies reported (Tommasone et al., 2019, and references therein), no covalent bonding is ever observed, the absence of charged groups and aromatic ring structures in simple sugars limiting interactions to hydrophobic sites and hydrogen bonding. In our simulations of the FG structure, some H-bonds are observed to form, and last for a substantial amount of time, typically between the hydroxyl OH oxygen of a mannose, and the phosphate oxygen O2P of the DNA backbone. Figure 6 depicts one example of such a bonding structure, implicating three consecutive guanines and two mannoses, which make up 4 H-bonds. (Note that the distinction between the two backbone oxygens–one of which should be doubly-bonded to the central P atom of the ${PO}_{4}^{-}$ group–is purely geometrical, since the two O have the same bond length and charge, in both the CHARMM and AMBER force fields.) None of the currently available glycan force fields are optimised for interaction with nucleic acids, therefore such bonding structures must be taken with caution; however, they are observed to occur always with the same repeated arrangement, which suggests it could not be a chance occurrence. Such observations open up a whole new field of investigation, and will certainly deserve further attention.

To have a more quantitative appraisal of the energy change associated with the aptamer-protein interaction, molecular contact surfaces were estimated with the PDBePISA web utility (Krissinel and Henrick, 2007), by using the standard rolling-sphere method with 1.4 Å probe radius. The apta2-S complex has 18.6 nm^2^ of contact surface with the S1 domain; the complex with the shorter apta1 has a correspondingly smaller contact surface of 10.7 nm^2^. PISA also provides an estimate of the solvation free energy Δ*G*_*s*_, by taking the difference between the isolated and interfaced atomic structures of the different fragments; such a value can be taken as a first estimate of the interfacial adhesion between the aptamer and the protein, however noting that the as-calculated value does not include the H-bonds energy. We thus obtained a Δ*G*_*s*_ = −24 ± 1 kcal/mol for the apta2, and Δ*G*_*s*_ = −14 ± 1 kcal/mol for the apta1. Furthermore, the extra H-bonding interaction of apta2 with the N subdomain of the adjacent monomer adds 17.5 nm^2^ of contact surface, with a corresponding extra contribution to the free energy of Δ*G*_*s*_ = −10.4 kcal/mol.

It is worth noting that both the DNA aptamers used in the present study appear to contact the S1 domain in regions adjacent to the ACE2 small binding area, and likely could interfere with the ACE2-RBD interaction. The strong bonding interaction of DNA aptamers with the RBD and N subdomains of the S-protein (as indicated by the respective Δ*G*_*s*_) leads to severe mechanical deformations of the latter: many elements of the protein are destructured from helix and sheet to a disordered coil, and lead to a much more loose contact at the RBD region (see below).

### 3.2 Free-Energy of Opening of the S1 Domain

**FIGURE 7:**
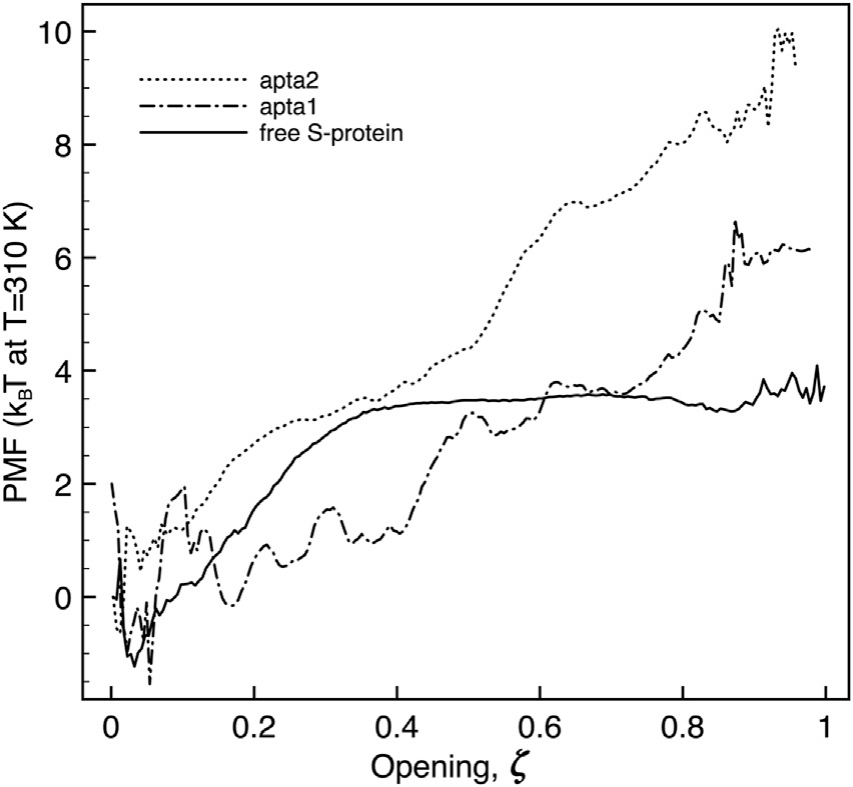
Plot of the potential of mean force extracted along the opening pathway *ζ*, for the free S-protein (full line), and the S-protein with one DNA aptamer docked (dashed lines). The free energy is in all cases estimated by the difference between the maximum and minimum value of the PMF along the reaction coordinate.

The umbrella sampling study allowed us to obtain the free-energies and the kinetic barriers for the S1-RBD subdomain going from the closed to the open configuration. Although this part of the study was carried out by a simplified free-energy method, we believe the results may nevertheless shed some light on the process, at least qualitatively. As detailed in the Methods section above, we defined a putative reaction coordinate *ζ* along the shortest path connecting the two extreme experimental configurations, and traced the potential of mean force (PMF). The reaction coordinate is normalized to [0, 1], corresponding to a physical motion of about 1.2 nm of the center of mass of the RBD subdomain of S1 (residues 319–541) with respect to the center of mass of the N subdomain (residues 14–305). Sample snapshots of the intermediate states are shown in Supplementary Figure S2 of Supplementary Material. The free energy difference between the open and closed conformations of the FG protein is in both cases estimated by taking the difference between the minimum and the maximum of the PMF all along the *ζ* coordinate. Figure 7 reports the plot of the PMF for the free S-protein, and for the two aptamer-protein configurations. It is observed that for the free protein (full curve) the transition from close to open goes through a small free energy barrier Δ*G*_*t*_ of just about 4.5 *k*_*B*_ *T* (the subscript “t” stands for “transition”), and proceeds without further barriers at constant energy from *ζ* ≃0.35 to 1. The initial barrier is likely associated with the unfolding of the “front” loop of the RBD (residues 465–495, see red arrow in Supplementary Figure S2B). Such findings confirm the experimental observation that the S1 domain can rather freely fluctuate between the two conformations, at physiological temperatures.

As hinted in Section 2.3 above, techniques such as metadynamics have been seldom used to study very large systems experiencing large hinge motions. A recent study (Gur et al., 2020) applied steered-MD to the opening of the S-protein; in that case, motion was provided by a force directed along a straight line connecting the center of mass of the RBD in the two extreme configurations. However, it appears that the motion of the RBD rather results from combination of rotations about different axes (Brotzakis et al., 2021). As a possible alternative, simplified methods such as rigid-body motions between two states are not at all new in the community (e.g., Ha and Loh, 2012; Tao et al., 2014) and several software packages exists to simulate the large-scale motion of domains and subdomains by similar methods (e.g., HingeProt http://bioinfo3d.cs.tau.ac.il/HingeProt, FATCAT https://fatcat.godziklab.org/). The method we designed in our work is also somewhat innovative, as it uses the finely spaced frames extracted from the rigid-body transformation between the closed and open states of the S1 subdomain, and applies MD thermalisation to each frame, in order to use the resulting pseudo-trajectory in the umbrella sampling free-energy method. The overlap of umbrella potential windows is extremely dense under such conditions (see Supplementary Figure S3 of Supplementary Material). An estimate of the statistical uncertainty of the calculated free energy plots by the “bootstrap” technique (Hub et al., 2010), is also shown in Supplementary Figure S4; the maximum fluctuation is about 15–20% at the end of the opening path. The more “noisy” free energy plot of Figure 7 is just due to this very fine spacing of the trajectory (about 50 times finer than usually done in umbrella sampling). That the final state may not look like a minimum is due to the fact that, with the aptamer attached, the final state is probably no longer a true “final state”. In fact, the radical modification of free-energy profiles upon binding to the receptor has been very recently suggested at least qualitatively by FRET studies (Lu et al., 2020b). A more conventional approach would have been to obtain the same transformation path by applying a directed force to a small group of atoms in the S1-RBD, however at the risk of producing unphysical distortions of the spike subdomain, given the typical speed of deformation in steered-MD (in the limit of applying the directed force to a larger and larger group of atoms, the rigid-body transformation is obviously recovered). Notably, in their already cited preprint Brotzakis et al., 2021 were able to reconstruct several realistic opening pathways, by introducing a complex interpolation procedure of cryo-EM images; however, such an advanced technique is well beyond the limited scope of the present work.

When a DNA aptamer is docked to the S1 domain, some important energetic changes indeed arise. As described above, the shorter **apta1** has a strong interaction with neighboring domains of the S-protein, its 5’ tail penetrating between the RBDs of two adjacent protomers. It appears here to affect significantly the opening kinetics (dash-dotted curve): the Δ*G*_*t*_ is increased to about 6 ± 1.5*k*_*B*_ *T*, with a substantial modification of the PMF profile. The opening follows two successive plateaux of about 2*k*_*B*_ *T* each, up to *ζ* = 0.4 and 0.8 respectively, to arrive at the fully opened conformation with a final slope.

The energetic response is similar, and more pronounced with the longer **apta2** docked to the S-protein. The Δ*G*_*t*_ jumps to 10.8 ± 2*k*_*B*_ *T*, thus signifying a relative reduction of the opening probability by about a factor 10^–3^ (ratio of the Δ*G*_*t*_ exponentials); the opening trajectory follows a nearly steady linear ramp, with a mild change of slope around *ζ* ∼ 0.5; a sharp minimum appears right before the final opening (however, such a feature could also be due to the numerical noise that affects the extremes at *ζ* ∼0 and ∼1 of all PMF plots, because of the somewhat reduced overlap of the sampling windows).

Despite some known limitations in interpreting PMF results (Darve, 2007), a steady slope in the PMF vs *ζ* plot may give an indication of the force needed to move from one conformation to another of the system. The average slope of about 10 *k*_*B*_ *T*/nm observed for apta2, should indicate an extra resistance to spontaneous switching of the S1-RBD subdomain from closed to open (with corresponding forces in the range 20–40 pN) once the DNA aptamer is docked. It may be worth noting that the energy- (or force-) displacement curves of Figure 7 could readily be subject to direct experimental testing by means of single-molecule force spectroscopy methods Ritort (2006); Landuzzi et al. (2020).

### 3.3 On the Binding of the Angiotensin Converting Enzyme-2 Receptor to S1-Receptor-Binding Subdomain Subdomain

In the light of the previous results, it may be now interesting to look at the possible interaction of the ACE2 receptor with the S1 domain, in such a partly-open conformation modified by the presence of the DNA aptamers. We ran a second series of docking simulations followed by a short MD thermal equilibration of the best docked structures, on the FG structure of the S-protein. Notably, even the most recent published experimental structures of the ACE2-spike interaction (Xiao et al., 2021) describe only small monosaccharides positioned at the putative sites of N-glycan binding, or are restricted only to the glycosylated RBD subdomain (Weekley et al., 2021). The present results should be taken as indicative of a generic system response, the atomic-scale details of the interactions being not yet comparable to any experimental data.

In its native conformation, ACE2 is known to make a large number of H-bonds at the RBD residues 498–501 with the *α*_1_-helix, plus bonds at LYS417, TYR453 and GLN474, according to the study by Yan et al. (2020); similarly, H-bonds at LEU455, ASN487, GLN493 and ASN501 are reported by Lan et al. (2020); further, weaker interactions (salt bridges, VdW) are also observed at some other residues in the range 440–505 of S1. A recent, detailed theoretical study (Wang et al., 2020) accurately described the H-bonding network, and also indicated the key role of hydrophobic interfaces and charge complementarity, in establishing the interaction of ACE2 with the RBD. In the first panel in Table 1, we report the H-bonds observed after a 50 ns MD annealing at *T* = 310 K of the FG S-protein, with the ACE2 receptor initially placed at the experimental configuration on the RBD in the open conformation (from J. Lan et al. (2020)); most of the experimentally identified bonds are maintained, plus a number of less strong ones; also, most of the H-bonds identified by Wang et al. (2020) are observed (although in that study, apparently no glycans were included in the MD simulations).

**TABLE 1:** Hydrogen bonds formed at the ACE2-S1 interface in the crystallographic experimental configuration (RCSB entry 6MJ0 (Lan et al., 2020), and in the “best binding” configurations from molecular dynamics simulations, starting with the **apta1** or **apta2** DNA aptamers docked to S1. Donor/acceptor species are labelled according to the AMBER99 atom codes (Ponder and Case, 2003); molecular structure data analysed by the PDBePISA utility (Krissinel and Henrick, 2007). **Experimental configuration (RCSB entry 6MJ0)**.

ACE2 side		Bond	S1 side	
Residue	Species	length (Å)	species	residue
GLN24^a^	OE1	2.69	ND2	ASN487
ASP30^b^	OD2	2.90	NZ	LYS417
GLN42	NE2	3.24	O	GLY446
GLN42	NE2	2.79	OH	TYR449
ASP38	OD2	2.69	OH	TYR449
TYR83	OH	2.79	OD1	ASN487
TYR83	OH	3.54	OH	TYR489
GLU35	OE2	3.50	NE2	GLN493
TYR41	OH	2.71	OG1	THR500
TYR41	OH	3.67	N	ASN501
GLU37	OE2	3.46	OH	TYR505
LYS353^c^	NZ	3.08	O	GLY496
LYS353	O	2.78	N	GLY502
ARG393	NH2	3.73	OH	TYR505
^*a*^ *terminal region.*				
^*b*^ *central region.*				
^*c*^ *beta-turn region of ACE2.*				

Best-binding configuration from docking with aptamer 1				
ACE2 side		Bond	S1 side	
Residue	species	length (Å)	species	Residue
LYS31	HZ3	1.93	OH	TYR453
HIS34	NE2	3.28	O	PHE456
SER19	OG	2.67	OE1	GLU471
ASP30	O	2.19	HE22	GLN493

Best-binding configuration from docking with aptamer 2				
ACE2 side		Bond	S1 side	
Residue	species	length (Å)	species	residue
GLN24	OE1	2.19	HD22	ASN487
TYR83	OH	3.42	N	TYR489

However, after binding the DNA aptamers, the adhesion capability of ACE2 to the open conformation of the S-protein is clearly reduced. In a first attempt, we contacted the ACE2 receptor to the RBD of the S-protein with apta2 taken in the final stage of the opening pathway, by just rigidly shifting the coordinates of ACE2 according to the experimental structure. Due to the presence of the aptamer, the contact surface area decreases from 8.4 to 6.2 nm^2^; bonding is also much affected, the number of H-bonds being reduced from 14 to 5, after losing contact between the S1 loop and the C-terminal of the *α*_1_-helix; the total free energy Δ*G* estimated by the PDBePISA method (also including the contribution from H-bonds and salt bridges) goes from −10.81 to −2.3 kcal/mol. However, the most notable information that comes from this rigid-shift superposition, is that the ACE2 sterically conflicts with the DNA aptamer over a large region, so that the contact structure of the ACE2-S-DNA complex must necessarily be modified upon the mutual interaction.

**FIGURE 8:**
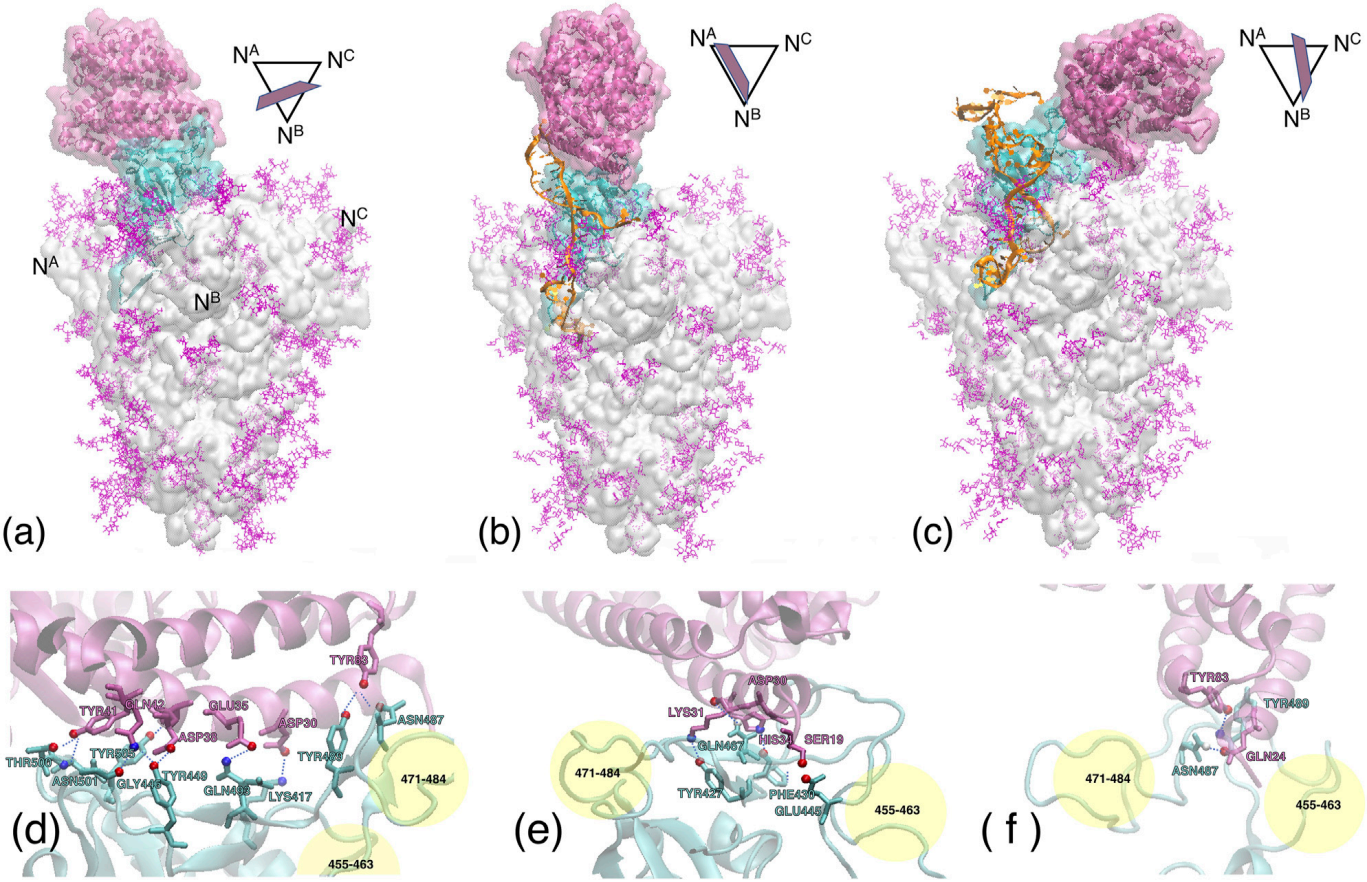
**Upper panel** Contact regions between the ACE2 receptor (mauve ribbons and transparent surface), the S1 subdomain (cyan ribbons and surface), and the DNA aptamer (orange ribbons). The remaining of the whole S-protein trimer is shown as a light grey transparent surface, with the glycans in purple. **(A)** Experimental configuration 6M0J after 50 ns MD equilibration. **(B)** MD simulation with the apta1. **(C)** MD simulation with the apta2. The central axis of the S-protein trimer is oriented vertically. The symbols above/right of each figure depict the approximate orientation of the *α*_1_-helix of ACE2, with respect to the cross section of the S-protein [the vertices indicate the N-terminals of each protomer, also reported in the panel (a)]. **Lower panel** Hydrogen bonds formed at the ACE2/RBD interface, for the experimental configuration **(D)** (only the central region indicated, see Table 1); apta1 **(D)**; and apta2 **(F)**. The yellow spheres approximately indicate the regions of residues 455–463 and 471–484 of the RBD, to provide a relative orientation of the lower figures with respect to the panel above.

Therefore, in a second step we performed a new series of docking runs, always using the HADDOCK web server. Also in this case, we ran different dockings by restricting the interaction of ACE2 with different portions of the RBD, and a larger run extended to the whole RDB; the configurations with the best score were then subject to force relaxation and a short, 50 ns MD annealing at *T* = 310 K. Cluster analysis of the resulting trajectory revealed the average binding configuration of ACE2 to the S-protein in the presence of either one of the two aptamers. Figure 8 shows in the upper panel the large-scale configurations of the ACE2 and S-protein system, in the pristine experimental structure after 50 ns of MD 1); upon interacting with the apta1 aptamer 2); and with the apta2 aptamer 3). It can be noted that the presence of the DNA strongly interferes with the ACE2 contact: the receptor is forced to turn by ∼90 deg about the central axis of the protein with the apta1, and it also gets inclined by ∼45 deg with respect to the central, vertical axis in the presence of the apta2, which sets up an extended steric protection of the RBD of the S-protein. Energy and surface results are summarized in Table 2: the contact surface between ACE2 and S1 is reduced to 7.6 nm^2^ with the apta1 and to 5.9 nm^2^ with the apta2; the total Δ*G* is reduced to −6.4 and −8.7 kcal/mol, respectively.

**TABLE 2:** Summary of free energy calculations with the PDBePISA utility (Krissinel and Henrick, 2007). Δ*G*_*s*_, Δ*G* values in kcal/mol.

Configuration	Contact	Δ*G*_*s*_	Hydrogen	Salt	Δ*G*
	Area (nm^2^)	Solvation	Bonds	Bridges	(Total)
Experimental	8.43	−4.5	14	1	−10.81
rigid shift/apta2	6.22	0.0	5	1	−2.35
docking/apta1	7.64	−2.2	4	1	−6.41
docking/DNA	3.57	−5.0	—	—	−5.00
docking/apta2	5.92	−7.2	2	4	−8.68

Compared to the abundant H-bonds of the experimental configuration without DNAs (see again Table 1), a much smaller number of H-bonds is formed by ACE2 with the RBD, in the presence of the aptamers. The receptor is still able to find the main binding region of the RBD, however the number of H-bonds is reduced from about 14 to just four for apta1, and merely two for apta2; almost no bonds survive from the experimental configuration, except for the TYR83-TYR489 in the apta2 case; the H-bonding region is now restricted to the fragment 427–467 of the RBD, and the beta-turn region (residues 353–393) of ACE2 makes no contact with the RBD; in particular, the salt bridge between ASP30 and LYS417 disappears. In the lower panel of Figure 8 we zoom on the contact region between the ACE2 receptor (mauve ribbons) and the RBD domain (cyan ribbons), in the experimental conformation **(d)**, and in the MD simulations including the apta1 **5)** and apta2 **(f)**; the residues implicated in H-bonds are highlighted with sticks, and joined by dashed lines. By comparison with the experimental adhesion structure in (a), it can be seen that the presence of the aptamers has the double effect of: 1) deforming the binding site, in particular by extracting the two loops 455–463 and 471–484 of the RBD (indicated by yellow shading in 4) and (f)), and 2) of disrupting some of the beta-sheets of these loops into disordered structures; the RBD interacts only with the *α*_1_-helix of the ACE2 receptor, which remains on the periphery of the binding surface with a much limited interaction. In either case 5) and (f), the pose of ACE2 is largely rotated with respect to the experimental, aptamer-free interaction (d), and the contact region only partly overlaps with the original one. In particular, the N-terminal and the beta-turn regions of ACE2 have lost any contact with the RBD.

Last but not least, it is worth noting that in the case of apta1 also a rather strong interaction is observed between ACE2 residues ASP67, LYS114, ASN63, ASN64, ASN121 (all charged or polar residues) and the protruding 5’ hairpin loop of the DNA aptamer (nucleotides 17–21), with an additional negative Δ*G*_*s*_ = −5 kcal/mol and an extra contact surface of 3.6 nm^2^, despite a lack of H-bonds or salt/disulfide bridges. The sum of the adhesion energy with ACE2 and the aptamer gives a Δ*G* = −11.41 kcal/mol, which translates into a factor 2 increase in the affinity with respect to the DNA-free interaction. (No contact with DNA is observed for the case of apta2, which keeps the ACE2 more far from the central region of RBD.) Such conformations with the receptor doubly bonded to a DNA aptamer and partly to the S1-RBD subdomain, are in principle very interesting. In a scenario in which aptamers are administered to a virus-infected ensemble of cells, such configurations successfully compete with, and preclude furthering of, the interaction between the cell receptors and the viral S-proteins, contributing to hamper the very early stages of the membrane fusion process.

## 4 Discussion and Conclusion

The spheroidal surface of the SARS-CoV-2 virus is decorated with a large density of copies of the transmembrane spike glycoprotein (S-protein), its three protomers being composed of two major S1 and S2 catalytic domains, plus other structural regions. As it is becoming clear from the recent literature (Walls et al., 2020), coronavirus entry in the host cell requires a concerted action of the receptor binding at the S1-RBD domain (typically, the receptor ACE2 present at the surface of most human cells), and the subsequent proteolytic processing of the S1-S2 link (also susceptible to furin cleavage), to allow the fusion domain S2 to initiate the fusion process between the virus and cell membranes (Shang et al., 2020). The S1 domain is experimentally found in two conformations: a “closed” one, in which the receptor binding sites (RBD) are inaccessible to ACE2, and an “open” one, in which ACE2 can effectively bind one S-protein from the virus. Both cryo-microscopy and X-ray diffraction data have shown that the S-protein protomers fluctuate between these two conformations with about 50/50 occupation probability (Walls et al., 2020; Wrapp et al., 2020). In our study we analyzed the interaction of two experimentally identified DNA aptamers (Song et al., 2020) with the whole trimeric structure of the S-protein, instead of focusing just on the very small binding regions as is typically done both in experimental and molecular docking studies. This more conservative and extensive choice allowed to reach some important conclusions, as detailed in the following.

One possible way in which aptamers could act as therapeutic devices would be to design their target nucleotide sequence so as to directly interfere with the receptor binding at the RBD. This was not entirely the case for the two experimentally identified aptamers used in this study. As we showed in the last Section 3.3, by means of docking and molecular dynamics simulations, their interaction with S1 occurs at a region very close to the RBD, enough to strongly modify the interaction site, and partly hide it from contact with the human ACE2 receptor. However, in order to exploit a more direct blocking effect, more precisely targeted aptamers should be identified experimentally.

On the other hand, another possibility is that aptamers may bind in such a way to limit, or even block the opening of the S1 domain, which is indeed the critical step to elicit the interaction with the cell receptor. Our finding that DNA aptamers with strongly specific interaction with the S1-RBD domain, can also interact with other subdomains of another protomer, thereby making a kind of “bridge” between pairs of adjacent protomers, induces important consequences. Results of free energy calculations by the umbrella sampling method, clearly demonstrate the possibility that the DNA aptamer bridging between two S monomers can actively block, or at least slow down considerably the opening of S1, which is the critical step to elicit the interaction with the cell receptor, thereby suppressing, or strongly reducing the receptor binding probability. The relatively high free energies of binding of the aptamers to the S-protein point to a very high (even ∼picomolar) sensitivity of the recognition mechanism.

In conclusion, we investigated by means of state-of-the-art protein docking and large-scale molecular dynamics simulations, the interaction of some experimentally identified DNA aptamers with the S-protein of SARS-CoV-2. We characterized in detail the DNA interaction with the fully glycosylated form of the S-protein in the closed conformation, identifying a network of hydrogen bonds that make for a high selectivity of the aptamer, as well as for a strong and stable adhesion. We showed that the DNA aptamers can bind efficiently to the designated receptor-binding domain (RBD) on one protomer of the S-protein, but also form and maintain stable bonds with other subdomains of adjacent protomers. Such an extended bonding interaction, actually impossible to deduce from the experimental measurements of generic binding affinity Song et al., 2020, is found to strongly restrain the opening of the RBD to the cell receptors, and should lead to a drastic reduction of the virus/cell binding efficiency.

Overall, the present results constitute a qualitative, rather than quantitative, suggestion for a novel biochemical interaction process, which may have important impact on the molecular mechanisms underlying viral invasion of the host cell. The fact that DNA aptamers are extremely selective, with sub-nanomolar sensitivity, very cheap to produce in large quantities, and extremely biocompatible with practically no adverse effects, since they have very little affinity for targets different from the one against which they are designed, make such findings a potential lead for a novel therapeutic concept.

## Data Availability

The datasets presented in this study can be found in online repositories. The names of the repository/repositories and accession number(s) can be found below: https://doi.org/10.6084/m9.figshare.12726896.v2

## References

[B1] Berendsen H. J. C. van der Spoel D. van Drunen R. (1995). GROMACS: A Message-Passing Parallel Molecular Dynamics Implementation. Comp. Phys. Commun. 91, 43–56. 10.1016/0010-4655(95)00042-e

[B2] Brotzakis Z. F. Lohr T. Vendruscolo M. (2021). Determination of Intermediate State Structures in the Opening Pathway of SARS-CoV-2 Spike Using Cryo-Electron Microscopy . Chem. Sci. 12, 9168–9175. 10.1039/D1SC00244A 34276947 PMC8261716

[B3] Casalino L. Gaieb Z. Goldsmith J. A. Hjorth C. K. Dommer A. C. Harbison A. M. (2020). Beyond Shielding: The Roles of Glycans in the SARS-CoV-2 Spike Protein. ACS Cent. Sci. 6, 1722–1734. 10.1021/acscentsci.0c01056 33140034 PMC7523240

[B4] Cheatham T. E. Case D. A. (2013). Twenty-five Years of Nucleic Acid Simulations. Biopolymers 99, 969–977. 10.1002/bip.22331 23784813 PMC3820278

[B5] Chen Z. Wu Q. Chen J. Ni X. Dai J. (2020). A DNA Aptamer Based Method for Detection of SARS-CoV-2 Nucleocapsid Protein. Virol. Sin. 35, 351–354. 10.1007/s12250-020-00236-z 32451881 PMC7246297

[B6] Cheng C. Dong J. Yao L. Chen A. Jia R. Huan L. (2008). Potent Inhibition of Human Influenza H5N1 Virus by Oligonucleotides Derived by SELEX. Biochem. Biophys. Res. Commun. 366, 670–674. 10.1016/j.bbrc.2007.11.183 18078808

[B7] Cleri F. Lensink M. F. Blossey R. (2020). DNA Aptamers Block the Receptor Binding Domain at the Spike Protein of SARS-CoV-2. chemrkiv. 10.26434/chemrxiv.12696173.v1 PMC8397481 34458322

[B8] Darmostuk M. Rimpelova S. Gbelcova H. Ruml T. (2015). Current Approaches in SELEX: An Update to Aptamer Selection Technology. Biotechnol. Adv. 33, 1141–1161. 10.1016/j.biotechadv.2015.02.008 25708387

[B9] Darve E. (2007). “Thermodynamic Integration Using Constrained and Unconstrained Dynamics,” in Free Energy Calculations: Theory and Applications in Chemistry and Biology. Editors Chipot C. Pohorille A. (Berlin: Springer), 46, 4. 10.1007/978-3-540-38448-9_4

[B10] Fadda E. Woods R. J. (2010). Molecular Simulations of Carbohydrates and Protein-Carbohydrate Interactions: Motivation, Issues and Prospects. Drug Discov. Today 15, 596–609. 10.1016/j.drudis.2010.06.001 20594934 PMC3936463

[B11] Famulok M. Mayer G. (2014). Aptamers and SELEX in Chemistry & Biology. Chem. Biol. 21, 1055–1058. 10.1016/j.chembiol.2014.08.003 25237853

[B12] Foloppe N. MacKerell, Jr. A. D. (2000). All-atom Empirical Force Field for Nucleic Acids: I. Parameter Optimization Based on Small Molecule and Condensed Phase Macromolecular Target Data. J. Comput. Chem. 21, 86–104. 10.1002/(sici)1096-987x(20000130)21:2<86::aid-jcc2>3.0.co;2-g

[B13] Grant O. C. Montgomery D. Ito K. Woods R. J. (2021). Analysis of the SARS-CoV-2 Spike Protein Glycan Shield Reveals Implications for Immune Recognition. Sci. Rep. 10, 14991. 10.1038/s41598-020-71748-7 PMC7490396 32929138

[B15] Gur M. Taka E. Yilmaz S. Z. Kilinc C. Aktas U. Golcuk M. (2020). Conformational Transition of SARS-CoV-2 Spike Glycoprotein between its Closed and Open States. J. Chem. Phys. 153, 075101. 10.1063/5.0011141 32828084

[B16] Ha J.-H. Loh S. N. (2012). Protein Conformational Switches: From Nature to Design. Chem. Eur. J. 18, 7984–7999. 10.1002/chem.201200348 22688954 PMC3404493

[B17] Hub J. S. de Groot B. L. van der Spoel D. (2010). g_wham-A Free Weighted Histogram Analysis Implementation Including Robust Error and Autocorrelation Estimates. J. Chem. Theor. Comput. 6, 3713–3720. 10.1021/ct100494z

[B18] Jang K. J. Lee N.-R. Yeo W.-S. Jeong Y.-J. Kim D.-E. (2008). Isolation of Inhibitory RNA Aptamers against Severe Acute Respiratory Syndrome (SARS) Coronavirus NTPase/Helicase. Biochem. Biophys. Res. Commun. 366, 738–744. 10.1016/j.bbrc.2007.12.020 18082623 PMC7092905

[B19] Jeddi I. Saiz L. (2017). Three-dimensional Modeling of Single Stranded DNA Hairpins for Aptamer-Based Biosensors. Sci. Rep. 7, 1178. 10.1038/s41598-017-01348-5 28446765 PMC5430850

[B20] Krissinel E. Henrick K. (2007). Inference of Macromolecular Assemblies from Crystalline State. J. Mol. Biol. 372, 774–797. 10.1016/j.jmb.2007.05.022 17681537

[B21] Lan J. Ge J. Yu J. Shan S. Zhou H. Fan S. (2020). Structure of the SARS-CoV-2 Spike Receptor-Binding Domain Bound to the ACE2 Receptor. Nature 581, 215–220. 10.1038/s41586-020-2180-5 32225176

[B22] Landuzzi F. Viader-Godoy X. Cleri F. Pastor I. Ritort F. (2020). Detection of Single DNA Mismatches by Force Spectroscopy in Short DNA Hairpins. J. Chem. Phys. 152, 074204. 10.1063/1.5139284 32087630

[B23] Lindahl E. Hess B. van der Spoel D. (2001). GROMACS 3.0: a Package for Molecular Simulation and Trajectory Analysis. J. Mol. Model. 7, 306–317. 10.1007/s008940100045

[B24] Lu M. Uchil P. D. Li W. Zheng D. Terry D. S. Gorman J. (2020b). Real-time Conformational Dynamics of Sars-Cov-2 Spikes on Virus Particles. Cell Host Microbe 28, 880–891. 10.1016/j.chom.2020.11.001 33242391 PMC7664471

[B25] Lu R. Zhao X. Li J. Niu P. Yang B. Wu H. (2020a). Genomic Characterisation and Epidemiology of 2019 Novel Coronavirus: Implications for Virus Origins and Receptor Binding. Lancet 395, 565–574. 10.1016/s0140-6736(20)30251-8 32007145 PMC7159086

[B26] MacKerell A. D. Bashford D. Bellott M. Dunbrack R. L. Evanseck J. D. Field M. J. (1998). All-Atom Empirical Potential for Molecular Modeling and Dynamics Studies of Proteins†. J. Phys. Chem. B 102, 3586–3616. 10.1021/jp973084f 24889800

[B27] Mallajosyula S. S. Jo S. Im W. MacKerell A. D. (2015). Molecular Dynamics Simulations of Glycoproteins Using CHARMM. Methods Mol. Biol. 1273, 407–429. 10.1007/978-1-4939-2343-4_25 25753723 PMC4537648

[B28] Nie C. Pouyan P. Lauster D. Trimpert J. Kerkhoff Y. Szekeres G. P. (2021). Polysulfates Block SARS‐CoV‐2 Uptake through Electrostatic Interactions. Angew. Chem. Int. Ed. 60, 15870–15878. 10.1002/anie.202102717 PMC8250366 33860605

[B29] Orellana L. (2019). Large-scale Conformational Changes and Protein Function: Breaking the In Silico Barrier. Front. Mol. Biosci. 6, 117. 10.3389/fmolb.2019.00117 31750315 PMC6848229

[B30] Pérez A. Luque F. J. Orozco M. (2012). Frontiers in Molecular Dynamics Simulations of DNA. Acc. Chem. Res. 45, 196–205. 10.1021/ar2001217 21830782

[B31] Pettersen E. F. Goddard T. D. Huang C. C. Couch G. S. Greenblatt D. M. Meng E. C. (2004). UCSF Chimera?A Visualization System for Exploratory Research and Analysis. J. Comput. Chem. 25, 1605–1612. 10.1002/jcc.20084 15264254

[B32] Ponder J. W. Case D. A. (2003). Force fields for Protein Simulations. Adv. Prot. Chem. 66, 27–85. 10.1016/s0065-3233(03)66002-x 14631816

[B33] Popenda M. Szachniuk M. Antczak M. Purzycka K. J. Lukasiak P. Bartol N. (2012). Automated 3D Structure Composition for Large RNAs. Nucl. Acids Res. 40, e112. 10.1093/nar/gks339 22539264 PMC3413140

[B34] Ritort F. (2006). Single-molecule Experiments in Biological Physics: Methods and Applications. J. Phys. Condens. Matter 18, R531–R583. 10.1088/0953-8984/18/32/r01 21690856

[B35] Shang J. Ye G. Shi K. Wan Y. Luo C. Aihara H. (2020). Structural Basis of Receptor Recognition by SARS-CoV-2. Nature 581, 221–224. 10.1038/s41586-020-2179-y 32225175 PMC7328981

[B36] Smithgall M. C. Dowlatshahi M. Spitalnik S. L. Hod E. A. Rai A. J. (2020). Types of Assays for SARS-CoV-2 Testing: A Review. Lab. Med. 51, e59–e65. 10.1093/labmed/lmaa039 32657343 PMC7454768

[B37] Song Y. Song J. Wei X. Huang M. Sun M. Zhu L. (2020). Discovery of Aptamers Targeting the Receptor-Binding Domain of the SARS-CoV-2 Spike Glycoprotein. Anal. Chem. 92, 9895–9900. 10.1021/acs.analchem.0c01394 32551560

[B38] Tao P. Sodt A. J. Shao Y. König G. Brooks B. R. (2014). Computing the Free Energy along a Reaction Coordinate Using Rigid Body Dynamics. J. Chem. Theor. Comput. 10, 4198–4207. 10.1021/ct500342h PMC4196739 25328492

[B39] Tommasone S. Allabush F. Tagger Y. K. Norman J. Köpf M. Tucker J. H. R. (2019). The Challenges of Glycan Recognition with Natural and Artificial Receptors. Chem. Soc. Rev. 48, 5488–5505. 10.1039/c8cs00768c 31552920

[B40] van Zundert G. C. P. Rodrigues J. P. G. L. M. Trellet M. Schmitz C. Kastritis P. L. Karaca E. (2016). The HADDOCK2.2 Web Server: User-Friendly Integrative Modeling of Biomolecular Complexes. J. Mol. Biol. 428, 720–725. 10.1016/j.jmb.2015.09.014 26410586

[B41] Walls A. C. Park Y.-J. Tortorici M. A. Wall A. McGuire A. T. Veesler D. (2020). Structure, Function, and Antigenicity of the SARS-CoV-2 Spike Glycoprotein. Cell 181, 281–292. 10.1016/j.cell.2020.02.058 32155444 PMC7102599

[B42] Wang Y. Liu M. Gao J. (2020). Enhanced Receptor Binding of SARS-CoV-2 through Networks of Hydrogen-Bonding and Hydrophobic Interactions. Proc. Natl. Acad. Sci. USA 117, 13967–13974. 10.1073/pnas.2008209117 32503918 PMC7322019

[B43] Weekley C. M. Purcell D. F. J. Parker M. W. (2021). SARS-CoV-2 Spike Receptor-Binding Domain with a G485R Mutation in Complex with Human ACE2. bioRxiv. 10.1101/2021.03.16.434488

[B44] Woo H. Park S. J. Choi Y. K. Park T. Tanveer M. Cao Y. (2020). Developing a Fully Glycosylated Full-Length SARS-CoV-2 Spike Protein Model in a Viral Membrane. J. Phys. Chem. B 124, 7128. 10.1021/acs.jpcb.0c04553 32559081 PMC7341691

[B45] Wrapp D. Wang N. Corbett K. S. Goldsmith J. A. Hsieh C.-L. Abiona O. (2020). Cryo-EM Structure of the 2019-nCoV Spike in the Prefusion Conformation. Science 367, 1260–1263. 10.1126/science.abb2507 32075877 PMC7164637

[B46] Xiao T. Lu J. Zhang J. Johnson R. I. McKay L. G. A. Storm N. (2021). A Trimeric Human Angiotensin-Converting Enzyme 2 as an Anti-sars-cov-2 Agent. Nat. Struct. Mol. Biol. 28, 202–209. 10.1038/s41594-020-00549-3 33432247 PMC7895301

[B47] Xu J. Zhao S. Teng T. Abdalla A. E. Zhu W. Xie L. (2020). Systematic Comparison of Two Animal-To-Human Transmitted Human Coronaviruses: SARS-CoV-2 and SARS-CoV. Viruses 12, 244. 10.3390/v12020244 PMC7077191 32098422

[B48] Yan R. Zhang Y. Li Y. Xia L. Guo Y. Zhou Q. (2020). Structural Basis for the Recognition of SARS-CoV-2 by Full-Length Human ACE2. Science 367, 1444–1448. 10.1126/science.abb2762 32132184 PMC7164635

[B49] Zadeh J. N. Steenberg C. D. Bois J. S. Wolfe B. R. Pierce M. B. Khan A. R. (2011). NUPACK: Analysis and Design of Nucleic Acid Systems. J. Comput. Chem. 32, 170–173. 10.1002/jcc.21596 20645303

[B50] Zhang Y. Skolnick J. (2005). TM-align: a Protein Structure Alignment Algorithm Based on the TM-Score. Nucleic Acids Res. 33, 2302–2309. 10.1093/nar/gki524 15849316 PMC1084323

[B51] Zuker M. Jacobson A. B. (1998). Using Reliability Information to Annotate RNA Secondary Structures. RNA 4, 669–679. 10.1017/s1355838298980116 9622126 PMC1369649

[B52] Zuker M. (2003). Mfold Web Server for Nucleic Acid Folding and Hybridization Prediction. Nucleic Acids Res. 31, 3406–3415. 10.1093/nar/gkg595 12824337 PMC169194

