# Supplementary material for: DNA Aptamers Block the Receptor Binding Domain at the Spike Protein of SARS-CoV-2
Source: Front Mol Biosci. 2021 Aug 12;8:713003. doi: 10.3389/fmolb.2021.713003 (PMC8397481; doi:10.3389/fmolb.2021.713003)

SUPPLEMENTARY MATERIALS

DNA aptamers block the receptor binding domain at the spike protein of SARS-CoV-2

by F. Cleri[1,*], M. F. Lensink[2] and R. Blossey[2]
[1]University of Lille, CNRS UMR8520 IEMN, Institut d'Electronique, Microélectronique et Nanotechnologie, and Department of Physics, F-59000 Lille, France
[2]University of Lille, CNRS UMR8576 UGSF, Uniteé de Glycobiologie Structurelle et Fonctionnelle, F-59000 Lille, France

Supplementary figure S1. 2D structures of the DNA aptamers **apta1** ((**a**), with 51 nucleotides) and **apta2** ((**b**), with 67 nucleotides) issued by `mfold`. Color codes refer to the ss-count, indicating the propensity for base-pairing, increasing from red (0), to green (0.33), to blue (0.67), to black(1). The complete color code and further explanations of the computational procedure can be found in Ref.[12], and at the url: https://unafold.rna.albany.edu (see section "Structure display and free energy determination"). Both (a) and (b) closely resemble the 2D-foldings from Ref.[9]. For apta2 we also show in **(c)** an alternate folding, which had a very close free energy; however, only folding (b) was used to generate the 3D structure. The following tables provide the individual contributions of each structural element to the overall folding free-energy ΔG.

Supplementary Table 1. Aptamer apta1 2D-folding (a).

Structural element	ΔG	Information
External loop	-1.70	1 ss bases + 2 closing helices
Stack	-2.20	External closing pair G31-C50
Stack	-2.40	External closing pair T32-A49
Stack	-3.30	External closing pair C33-G48
Stack	-2.10	External closing pair C34-G47
Helix	-10.00	5 base pairs
Hairpin loop	+6.20	Closing pair A35-T46
Stack	-2.10	External closing pair C1-G30
Stack	-2.10	External closing pair A2-T29
Stack	-3.40	External closing pair G3-C28
Stack	-2.10	External closing pair C4-G27
Stack	-2.20	External closing pair A5-T26
Helix	-11.90	6 base pairs
Interior loop	+1.20	External closing pair C6-G25
Stack	-3.30	External closing pair C10-G22
Helix	-3.30	2 base pairs
Hairpin loop	+4.40	Closing pair C11-G21

Supplementary Table 2. Aptamer apta2 2D-folding (b).

Structural element	ΔG	Information
External loop	-0.40	3 ss bases + 2 closing helices
Stack	-2.40	External closing pair C53-G66
Helix	-2.40	2 base pairs
Hairpin loop	+4.10	Closing pair G54-C65
Stack	-1.10	External closing pair A1-T50
Stack	-2.40	External closing pair T2-A49
Stack	-3.30	External closing pair C3-G48
Helix	-6.80	4 base pairs
Interior loop	+0.40	External closing pair C4-G47
Stack	-2.40	External closing pair G6-C45
Helix	-2.40	2 base pairs
Bulge loop	+4.50	External closing pair A7-T44
Stack	-3.40	External closing pair G13-C43
Stack	-2.10	External closing pair C14-G42
Stack	-2.10	External closing pair A15-T41
Stack	-3.40	External closing pair G16-C40
Helix	-11.00	5 base pairs
Hairpin loop	+5.90	Closing pair C17-G39

Supplementary Table 3. Aptamer apta2 2D-folding (c).

Structural element	ΔG	Information
External loop	-1.10	9 ss bases + 2 closing helices
Stack	-1.40	External closing pair C53-G60
Helix	-1.40	2 base pairs
Hairpin loop	+4.70	Closing pair G54-T59
Stack	-1.10	External closing pair A1-T50
Stack	-2.40	External closing pair T2-A49
Stack	-3.30	External closing pair C3-G48
Helix	-6.80	4 base pairs
Interior loop	+0.40	External closing pair C4-G47
Stack	-2.40	External closing pair G6-C45
Helix	-2.40	2 base pairs
Bulge loop	+4.50	External closing pair A7-T44
Stack	-3.40	External closing pair G13-C43
Stack	-2.10	External closing pair C14-G42
Stack	-2.10	External closing pair A15-T41
Stack	-3.40	External closing pair G16-C40
Helix	-11.00	5 base pairs
Hairpin loop	+5.90	Closing pair C17-G39

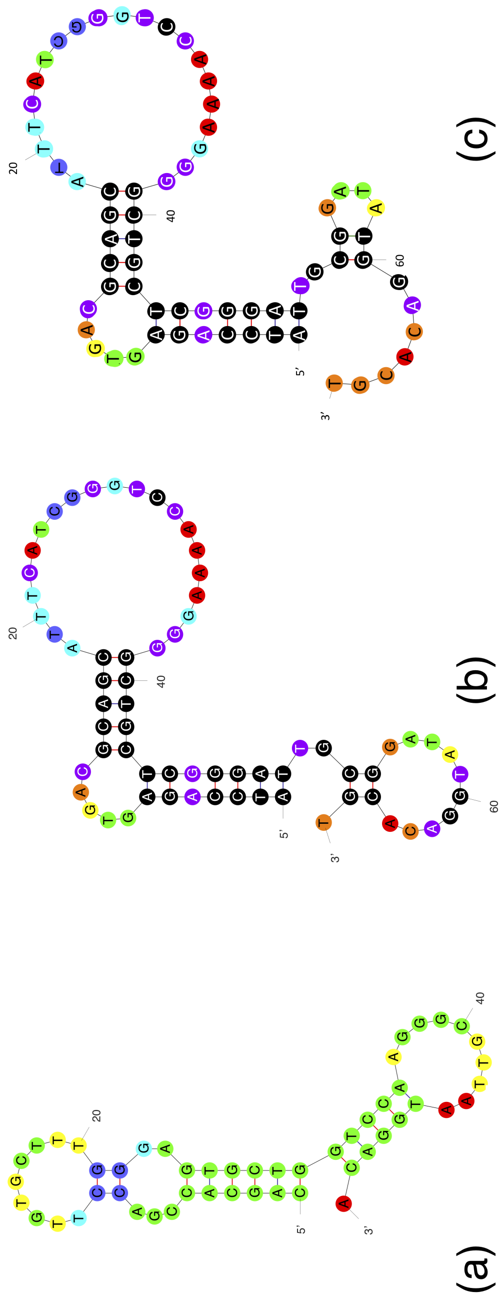

Supplementary Figure 1

Supplementary figure S2. Sample snapshots along the opening path of the RBD, in contact with the DNA aptamer apta2. DNA in orange ribbons, RBD of protomer 1 in cyan, and NTD of protomer 2 in blue; the rest of the S-protein is shown as a grey surface in the background; glycans omitted for clarity. Panels (a) to (f) correspond to the reaction coordinate ζ=0, 0.17, 0.35, 0.55, 0.7, 0.95, respectively.

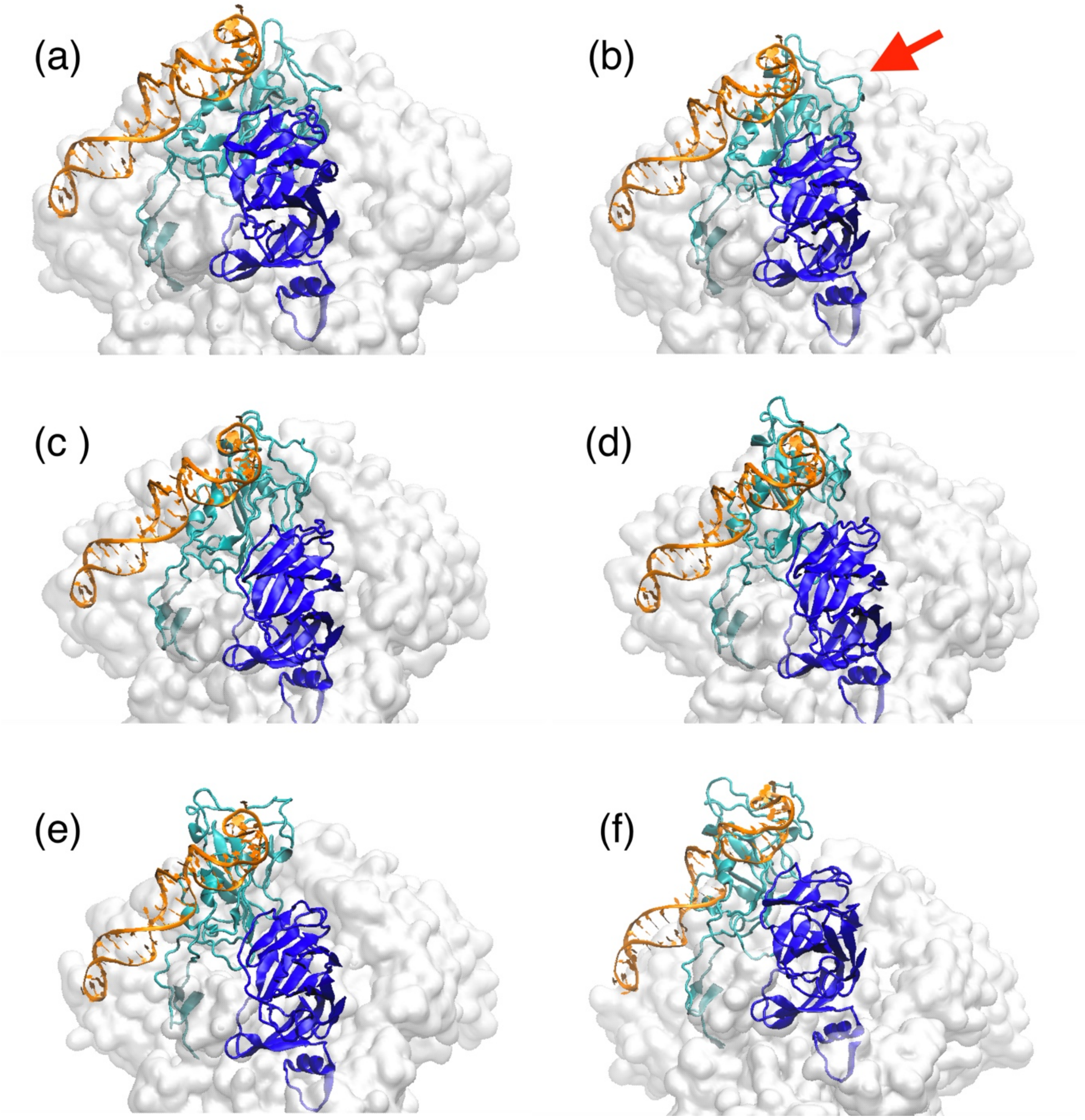

Supplementary figure S3. Weighted histogram analysis of the overlap between umbrella windows in the reaction coordinate ζ=0 to 1 (real opening coordinate covering about 1.2 nm); count number in ordinates in arbitrary units. (a) PMF of apta1. (B) PMF of apta2. (See main text and Fig. 7 for the free-energy analysis.)

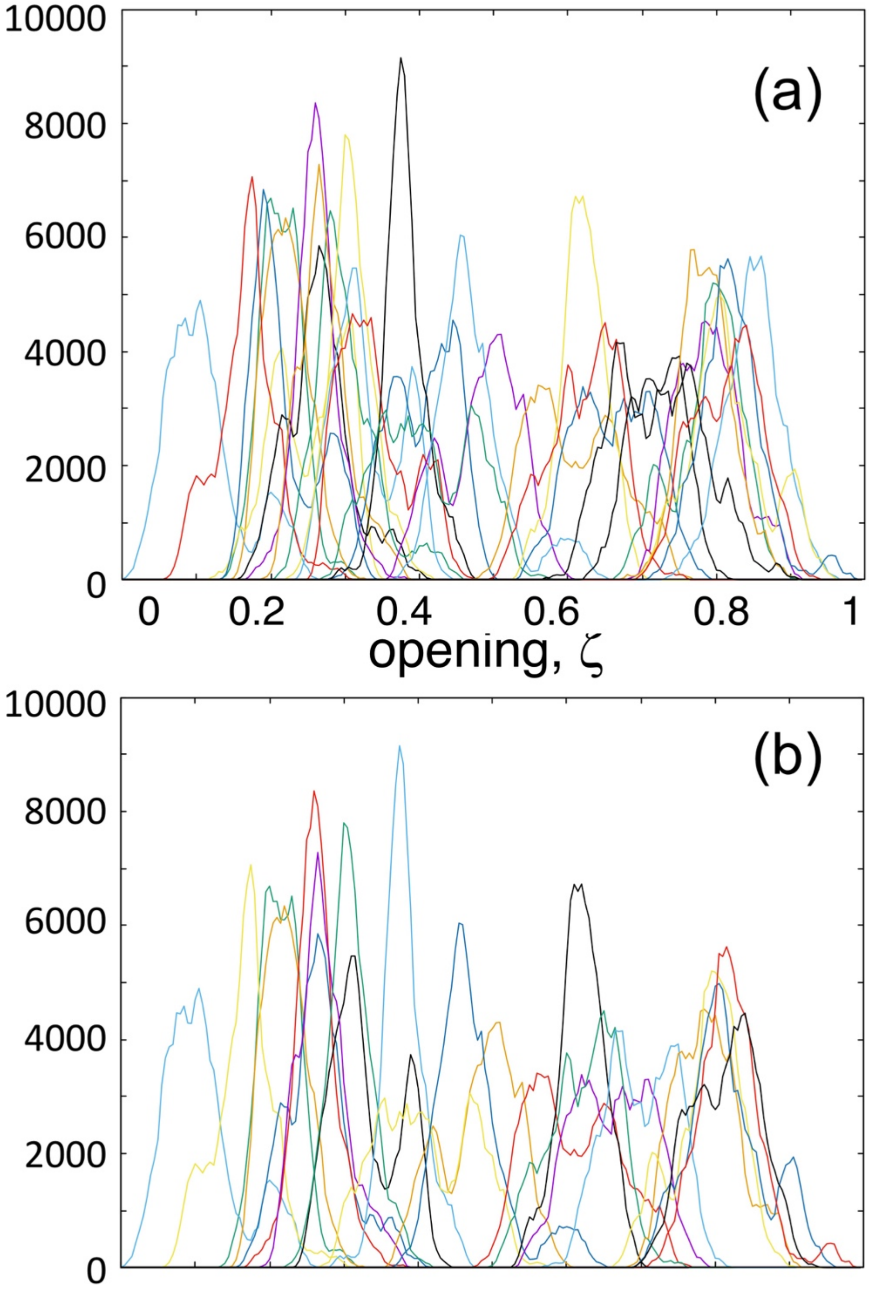

Supplementary figure S4. Statistical error estimate with bootstrap analysis from the GROMACS `wham` utility, for the free energy plots of DNA aptamers **apta1** and **apta2**, along the opening path of the S-protein RBD.

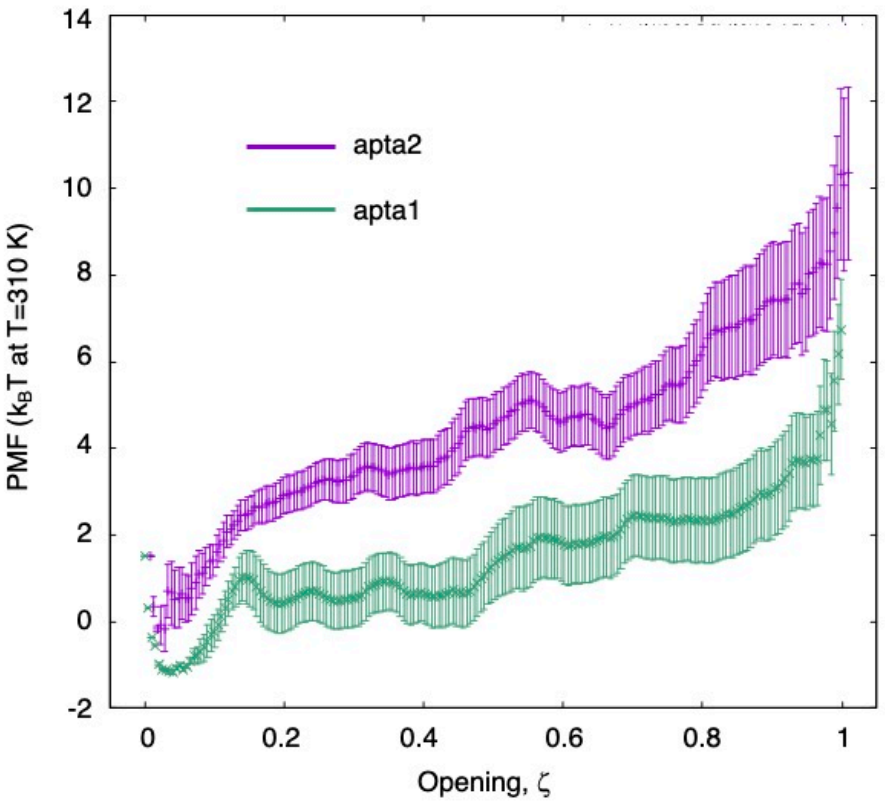

Supplement: Supplementary file 1 [file DataSheet1.PDF]
